# Arginine Kinase Activates Arginine for Phosphorylation by Pyramidalization and Polarization

**DOI:** 10.1021/acscatal.4c00380

**Published:** 2024-04-16

**Authors:** Fabio Falcioni, Robert W. Molt, Yi Jin, Jonathan P. Waltho, Sam Hay, Nigel G. J. Richards, G. Michael Blackburn

**Affiliations:** † Department of Chemistry, University of Manchester, Manchester M13 9PL, U.K.; ‡ ENSCO Inc., Melbourne, Florida 32940, United States; § Manchester Institute of Biotechnology, University of Manchester, Manchester M1 7DN, U.K.; ∥ School of Chemistry, Cardiff University, Park Place, Cardiff CF10 3AT, U.K.; ⊥ Foundation for Applied Molecular Evolution, Alachua, Florida 32615, United States; # School of Biosciences, University of Sheffield, Sheffield S10 2TN, U.K.

**Keywords:** arginine kinase, phosphorylation, pyramidalization, transition state, density functional theory

## Abstract

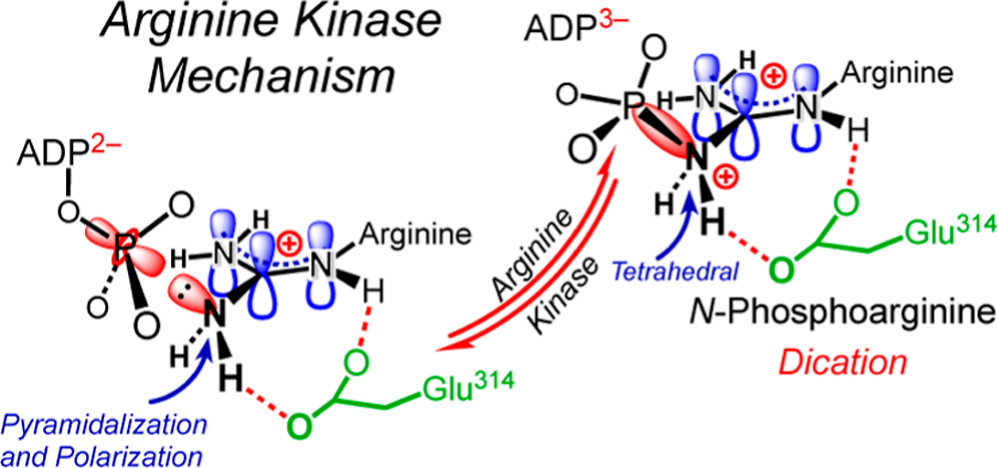

Arginine phosphorylation plays numerous roles throughout biology. Arginine kinase (AK) catalyzes the delivery of an anionic phosphoryl group (PO_3_^–^) from ATP to a planar, trigonal nitrogen in a guanidinium cation. Density functional theory (DFT) calculations have yielded a model of the transition state (TS) for the AK-catalyzed reaction. They reveal a network of over 50 hydrogen bonds that delivers unprecedented pyramidalization and out-of-plane polarization of the arginine guanidinium nitrogen (Nη2) and aligns the electron density on Nη2 with the scissile P–O bond, leading to in-line phosphoryl transfer via an associative mechanism. In the reverse reaction, the hydrogen-bonding network enforces the conformational distortion of a bound phosphoarginine substrate to increase the basicity of Nη2. This enables Nη2 protonation, which triggers PO_3_^–^ migration to generate ATP. This polarization–pyramidalization of nitrogen in the arginine side chain is likely a general phenomenon that is exploited by many classes of enzymes mediating the post-translational modification of arginine.

## Introduction

**Figure 1:**
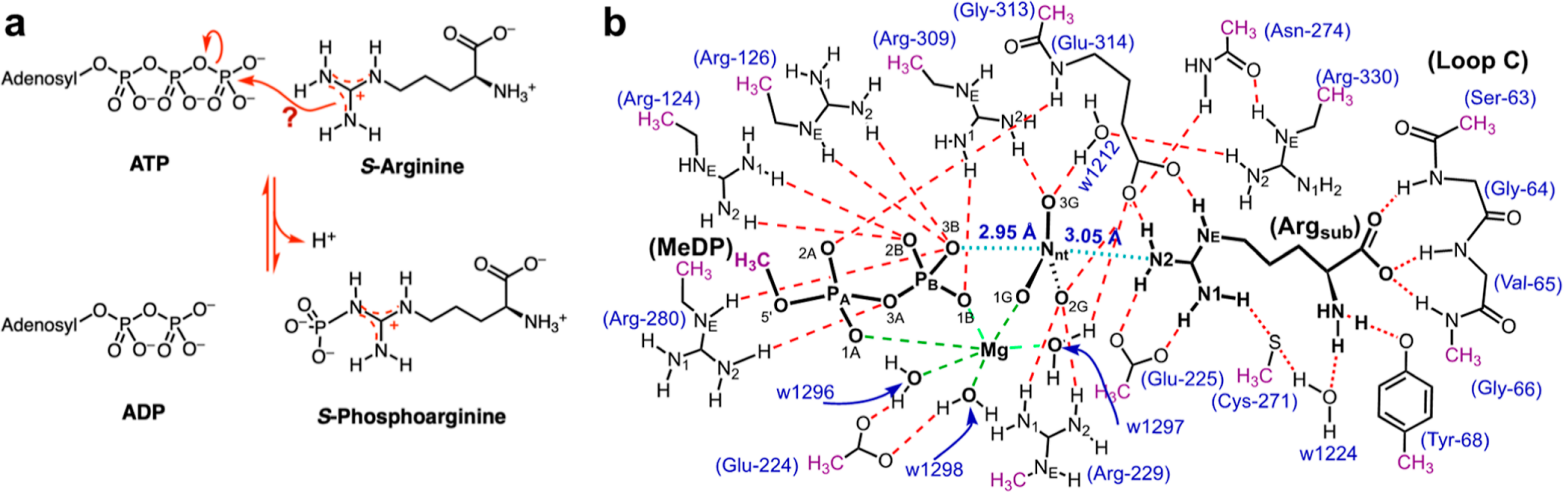
AK-catalyzed reaction and catalytic components. (a) Structures of reactants and products. Curly arrows indicate that the protonated arginine side chain acts as a nucleophile in the forward reaction via an unresolved mechanism. (b) Hydrogen bond network present in the active site model used to compute the structure of the AK·Mg·ADP·NO_3_^–^·arginine complex. This model, which is derived from an X-ray crystal structure (PDB: 1m15), comprises 17 catalytically relevant amino acids, the magnesium ion, and its 3 coordinating waters (indicated by green dashes). Atoms of reactants and the nitrate anion are shown in bold font; *r*_DA_ distances (bold, blue font) are those in the crystal structure 1m15; hydrogen bonds in the network are shown as red dashes (lengths not to scale); 3 peripheral waters and their associated hydrogen bonds are omitted for clarity, as are ionic charges.

Arginine phosphorylation is well-known as a physiological strategy for buffering ATP concentration.^1^ Phosphoarginine residues in proteins, however, also play a myriad of important functional roles in biology.^2,3^ Arginine kinase (AK, EC 2.7.3.3) catalyzes the reversible transfer of the γ-phosphate of Mg·ATP to the guanidinium moiety of free arginine (Figure 1a).^4^ In the forward direction, AK generates phosphoarginine and Mg·ADP; a reaction that is endergonic at pH 7.0 and 25 °C.^5^ Phosphorylation of the guanidinium side chain with Mg·ATP is a remarkable chemical transformation. For example, since the arginine side chain is protonated at physiological pH, phosphorylation requires a formally cationic group to act as a nucleophile, which is intrinsically difficult because of π-electron delocalization in the guanidium group. It is therefore surprising, given a wealth of experimental and structural information,^6−9^ that few molecular details are known concerning exactly how AK activates arginine side chains for reaction.

Efforts to develop a mechanistic model for AK action have also been informed by experimental studies on creatine kinase (CK), the evolutionary predecessor of AK.^10^ Mutagenesis studies on AK identify six essential arginines, two glutamates, and a cysteine thiolate as important for catalytic function.^7,11−13^ There remains, however, a lack of consensus concerning their roles in acid–base catalysis, ground-state (GS) destabilization, or induced fit contributions to catalysis.

Quantum chemistry calculations have provided important insights into the chemical mechanism and energetics of phosphoryl transfer to oxygen in biologically important transformations,^14−17^ including GTP hydrolysis^18−22^ and the formation of phosphosugars (Table S1).^23^ With the exception of phosphohistidine formation,^24^ which proceeds via nucleophilic attack of a lone pair in an orbital orthogonal to the aromatic π-electron system, there are no computational studies of enzyme-catalyzed phosphoryl transfer to nitrogen. Here we use density functional theory (DFT) to investigate the details of AK-catalyzed arginine phosphorylation. These calculations resolve the following key questions: (1) how does AK activate arginine to make a nucleophile from a trigonal planar nitrogen? (2) does phosphoryl transfer proceed via an associative or a dissociative TS? (3) can phosphagen kinase–nitrate complexes be employed to identify the actual TS for phosphoryl transfer to arginine? (4) is nitrate anion really a TSA for the phosphoryl group, PO_3_^–^? (5) at what stage is the proton released from the phosphoarginine product? We believe that our computational answers will be generally applicable to other biologically important enzymes that activate arginine side chains for reaction.^25^

## Results and Discussion

### Obtaining a Transition-State Model for AK-Catalyzed Phosphoryl Transfer

**Figure 2:**
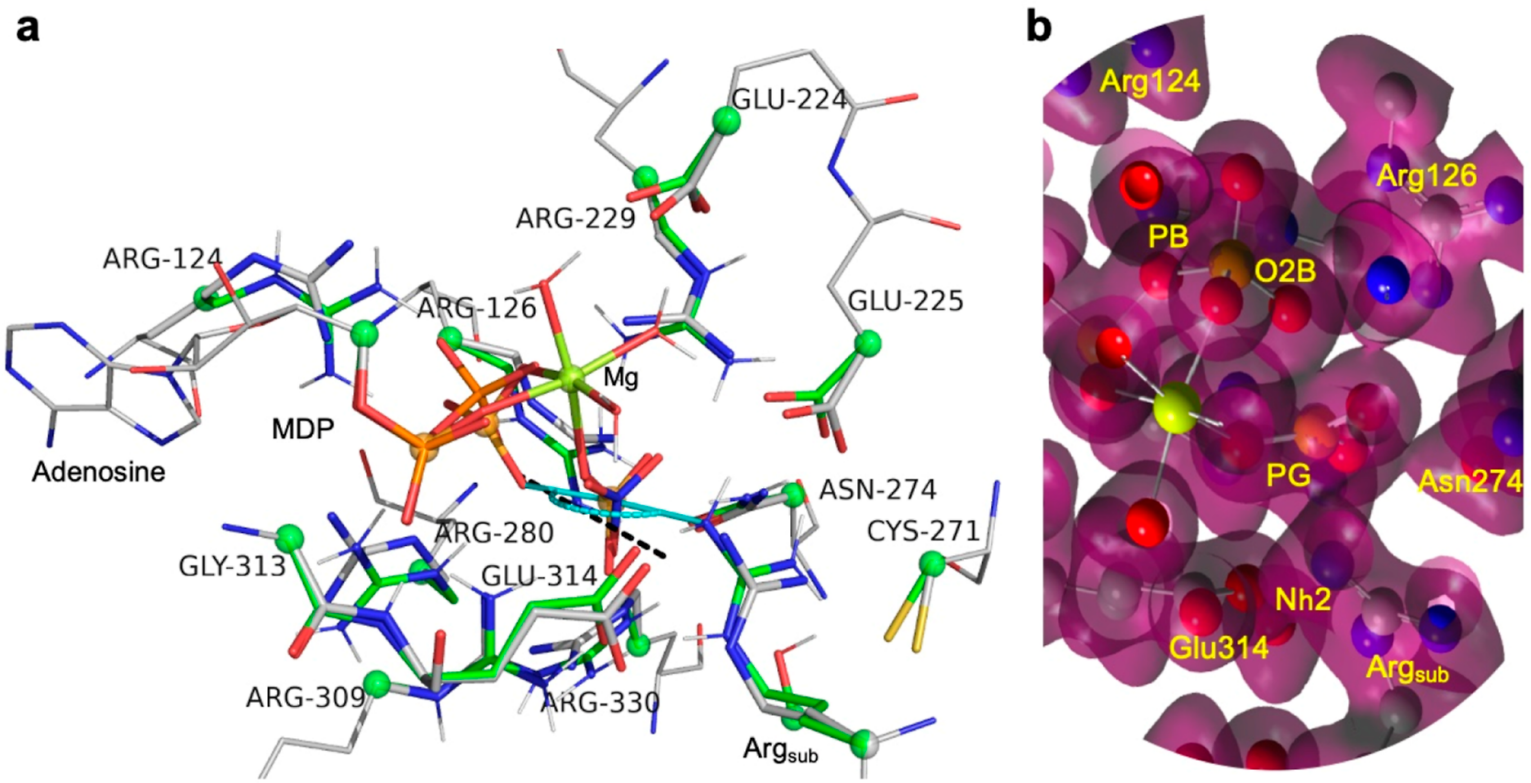
Comparing the calculated, small TS cluster model with the crystal structure of the AK·Mg·ADP·NO_3_^–^·arginine complex (PDB: 1m15). (a) AK·Mg·ADP·PO_3_^–^·Arg_sub_ (C–C bonds shown by green sticks) region has 14 “locked” carbons, including Arg_sub_ (green spheres). Water molecules in the two structures are omitted for clarity (Figure S2). The corresponding amino acid residues from the nitrate crystal complex (1m15, gray sticks) are shown complete beyond the fixed atoms (faint gray lines). *r*_DA_ from O3B to Nη2 is 4.56 Å, and the “in-line” angle is 169.3° for the computed TS, and 5.98 and 175.8° in the X-ray crystal structure (MDP, methyl diphosphate; phosphorus, orange spheres; magnesium, lemon sphere). (b) Section of the ED map for the core Mg·MeDP·PO_3_^–^·Arg_sub_ in the small TS model contoured at 0.40 e^–^ Å^–3^ (frontal density was removed for clarity).

We optimized the geometry of an active site cluster model constructed from the heavy atom coordinates in the X-ray crystal structure AK·Mg·ADP·NO_3_^–^·Arg_sub_ complex (PDB: 1m15).^6^ This initial cluster model (229 total atoms) had components from 13 amino acids, with 14 peripheral carbons being constrained to their crystallographic coordinates in the absence of protein residues surrounding the active site model. The model also contained 12 crystallographic waters that form key H-bond interactions and an additional two water molecules that were added to minimize artifactual charge density effects at the boundary of the model (chosen by criteria detailed in the Supporting Information). Energy minimization gave an optimized “AK-nitrate” model, which shows excellent agreement with the crystal structure (rmsd 0.32 Å for 87 nonwater, heavy atoms) (Figure S1). The network of core H-bonds, excluding those to peripheral waters in the model, is identical to those inferred from interatomic distances in the X-ray crystal structure (Figure 1b). Specifically, the guanidinium group of the substrate arginine donates five H-bonds to three amino acid acceptors in the model. We next replaced the NO_3_^–^ nitrogen by phosphorus and reoptimized the molecular geometry using standard methods^26^ to locate the TS geometry while retaining the peripheral carbons at their initial positions. The resulting TS cluster model contained 40 H-bonds and exhibited a donor–acceptor distance (*r*_DA_) of 4.56 Å (Figure 2a), considerably reduced from the value of 5.98 Å seen in the optimized “AK-nitrate” cluster model. Very importantly, we observed that the phosphoryl-acceptor nitrogen (Nη2) had changed shape in the TS, becoming pyramidal rather than remaining planar, as seen in the “AK-nitrate” model. As a result, a significant “bulge” of electron density (ED) is evident on Nη2 (contoured at 0.40 e^–^ Å^–3^) that points directly at PG in the transferring phosphoryl group (Figure 2b). Efforts to locate a dissociative TS all failed, even when the exact Hessian was calculated at every step of the TS search, as discussed in the Supporting Information.

### Calculated Energetics for AK-Catalyzed Phosphoryl Transfer

To establish whether our small TS cluster model was representative of the ensemble of states connecting reactants to products during AK-catalyzed phosphoryl transfer, we used intrinsic reaction coordinate (IRC) calculations to locate the associated AK·Mg·ATP·Arg_sub_ (“reactant”) ternary complex (Figure S3).^27^ The calculated energies of the cluster models for the reactant complex and TS gave an estimate of Δ*H* = 58 kJ mol^–1^ for the barrier associated with AK-catalyzed phosphoryl transfer from ATP (Figure S4). We note, however, that an IRC yields a structure that results from following one particular vibrational mode in the TS. As a result, the final reactant structure may not represent the true global minimum, because all other motions are ignored. Thus, we find that the γ-phosphate of ATP is not fully relaxed in the converged structure obtained from this IRC calculation, and our Δ*H* value is therefore best regarded as a lower bound.

The temperature dependence of the turnover number (*k*_cat_) measured for phosphoarginine formation catalyzed by a range of arginine kinases has been the subject of several experimental investigations (Table S2). Although there is considerable variation in the reported values of activation entropy and activation enthalpy, the activation free energies (Δ*G*^‡^) fall within the range 60–72 kJ mol^–1^. However, based on NMR relaxation measurements, it has been suggested that chemistry is not the rate-limiting step for the AK-catalyzed reaction of free arginine and Mg·ATP. Thus, the observed energy barrier may arise from a conformational transition in the N-terminal domain (NTD) during turnover rather than from the phosphoryl transfer step.^9^ If this is indeed the case, the free-energy barrier for the chemical step must be somewhat lower than or similar in magnitude^28^ to the values reported in Table S2. Moreover, because Δ*H* does not include entropic contributions, which are expected to increase the free-energy barrier, and as the error generally associated with DFT calculations can be large (±14 kJ mol^–1^),^29,30^ we consider our IRC-based barrier to be in reasonable agreement with the experiment.

### Enlarging the TS Cluster Model for AK-Catalyzed Phosphoryl Transfer

**Figure 3:**
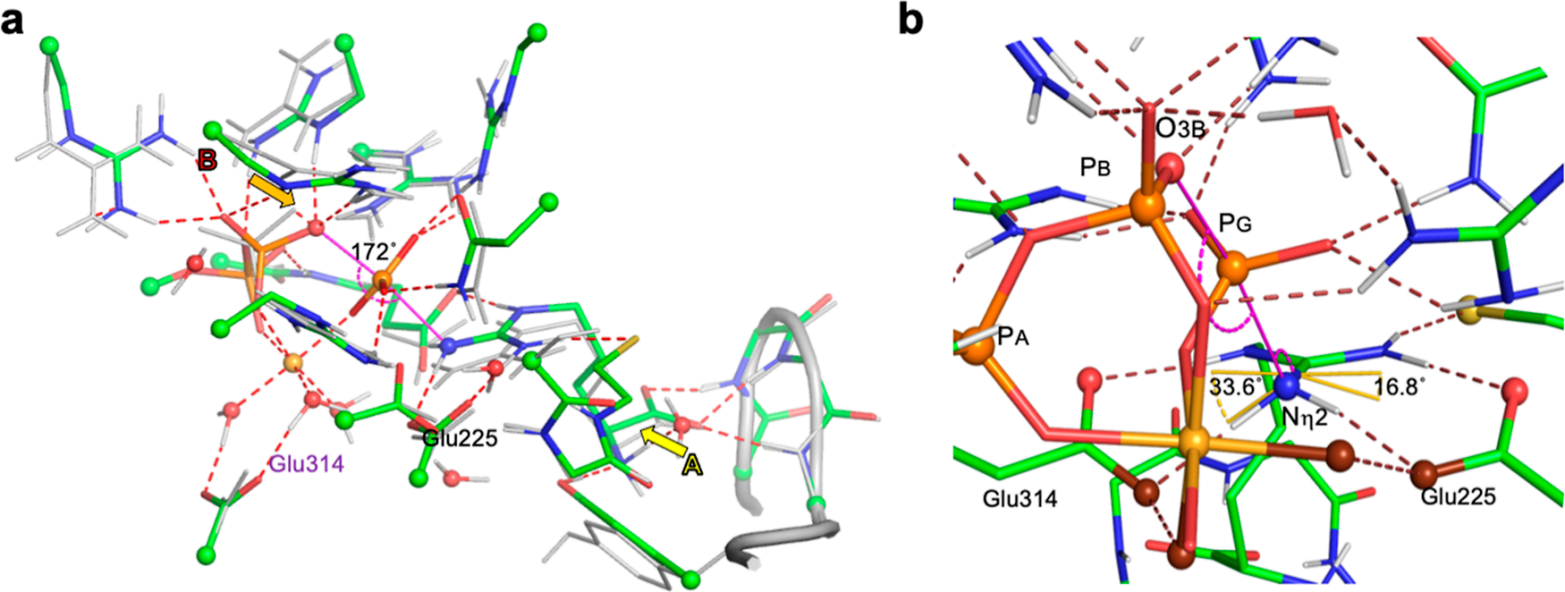
Enlarged TS cluster model for AK-catalyzed phosphoryl transfer. (a) Enlarged TS cluster model for AK (green sticks) aligned with the corresponding residues for the optimized reactant cluster model derived from 1m15 (silver sticks) with 16 fixed carbons (green spheres). The cartoon depicts atoms in the NTD loop comprising residues 61–68 (silver tube); H-bonds (red dashes) correspond to those in the QM TS cluster model (Figure 1b). Three waters coordinate the catalytic magnesium (orange sphere), and four waters interact with key protein residues (red spheres). The in-line angle is 172°, and *r*_DA_ is 4.66 Å (magenta bar). (b) Conformational detail of the Arg_sub_ guanidinium group showing H-bonding to Cys271, Glu225, and Glu314. Implicit pyramidalization of Nη2 is indicated by dihedral angles 33.6° to Glu314/OE2 and 16.8° to Glu225/OE2 (yellow arcs; oxygens, ruby spheres). In-line link O3B–PG–Nη2 (magenta bar) also shows an approach angle of PG to the guanidinium plane of 121° (magenta arc).

Concerned by the possibility that the *r*_DA_ value in the small TS cluster model might be incorrect because of our decision to constrain the butylguanidinium moiety representing Arg_sub_, we next built and optimized a second, larger TS cluster model (275 total atoms) that included all of the atoms in Arg_sub_ together with additional groups from residues 61–68 located in the NTD, which coordinate the α-amino and carboxylate moieties of Arg_sub_ (Figure 3a). Optimization of this larger cluster model again delivered a slightly unsymmetric, associative TS, with an *r*_DA_ of 4.66 Å, an in-line O–P–O angle of 172°, and axial PG–O3B and PG–Nη2 bond lengths of 2.21 and 2.47 Å, respectively.^1^ Remarkably, the larger TS cluster model contains a network of over 50 H-bonds (Table S3). Five are donated by the guanidinium moiety of Arg_sub_ with another five involving the α-amino and carboxylate groups. An additional 13 H-bonds are directed at the oxygens of Mg·ADP and the phosphoryl group undergoing transfer to arginine, with the catalytically important Mg^2+^ also coordinating the oxygens of O1A, O1B, and O1G (Figure 3b).

### Substrate-Based Conformational Changes in the Larger TS Cluster Model

Although atoms corresponding to the α-carbons of Ser63, Val65 and Tyr68 are constrained in the larger TS model, the additional interactions still permit Arg_sub_ to move from its original position (arrow A in Figure 3a) to bring Nη2 closer to the transferring phosphorus (PG). Complementing that shift, the substrate donor atom O3B moves 0.75 Å in-line toward PG (arrow B in Figure 3a) due to a 25° rotation of the O1A–PB bond, supported by H-bonds from a conformational repositioning of Arg124. The combined result of these conformational changes is an *r*_DA_ value of 4.66 Å, slightly longer than that seen in our small TS model but markedly shorter than the corresponding distance in the “AK-nitrate” structure. H-bonds also orient and polarize the guanidinium group of Arg_sub_. Critically, three H-bonds, from Nδ to Glu314 and from Nη1 to Glu225 and Cys271, are virtually in-plane and position Nη2 for in-line phosphoryl transfer (Figure 3b). In sharp contrast, the two H-bonds from acceptor nitrogen Nη2 to Glu225 and Glu314 are out-of-plane by 17 and 34° respectively, an out-of-plane distortion of NH–O bonds first observed by Cotton and co-workers in 1972.^32^ Notably, although Glu225 does not move significantly from its initial position in the “AK-nitrate” model, OE2 in the carboxylate of Glu314 shifts 1 Å to maintain its H-bond to Nη2 in the TS cluster model (Figure 3a). Together, these H-bonds define a TS in which Nη2 is pyramidalized by 47°, becoming oriented toward the transferring phosphorus to deliver a PG–Nη2–Cζ angle of 121°. Thus, the pyramidalization that is presumably needed to disrupt resonance stabilization in the guanidinium moiety and to increase the nucleophilicity of Nη2 is observed in both of our TS cluster models. We conclude that this out-of-plane (oop) distortion, facilitated by the extensive H-bond network, is a central feature of the catalytic mechanism of AK and, very likely, other phosphagen kinases. It is this remarkable distortion that converts a trigonal, planar delocalized guanidinium cation into a localized amino-formamidinium cation, allowing rehybridization of the Nη2 nitrogen to direct ED toward PG at the angle needed for reaction.^33^

**Figure 4:**
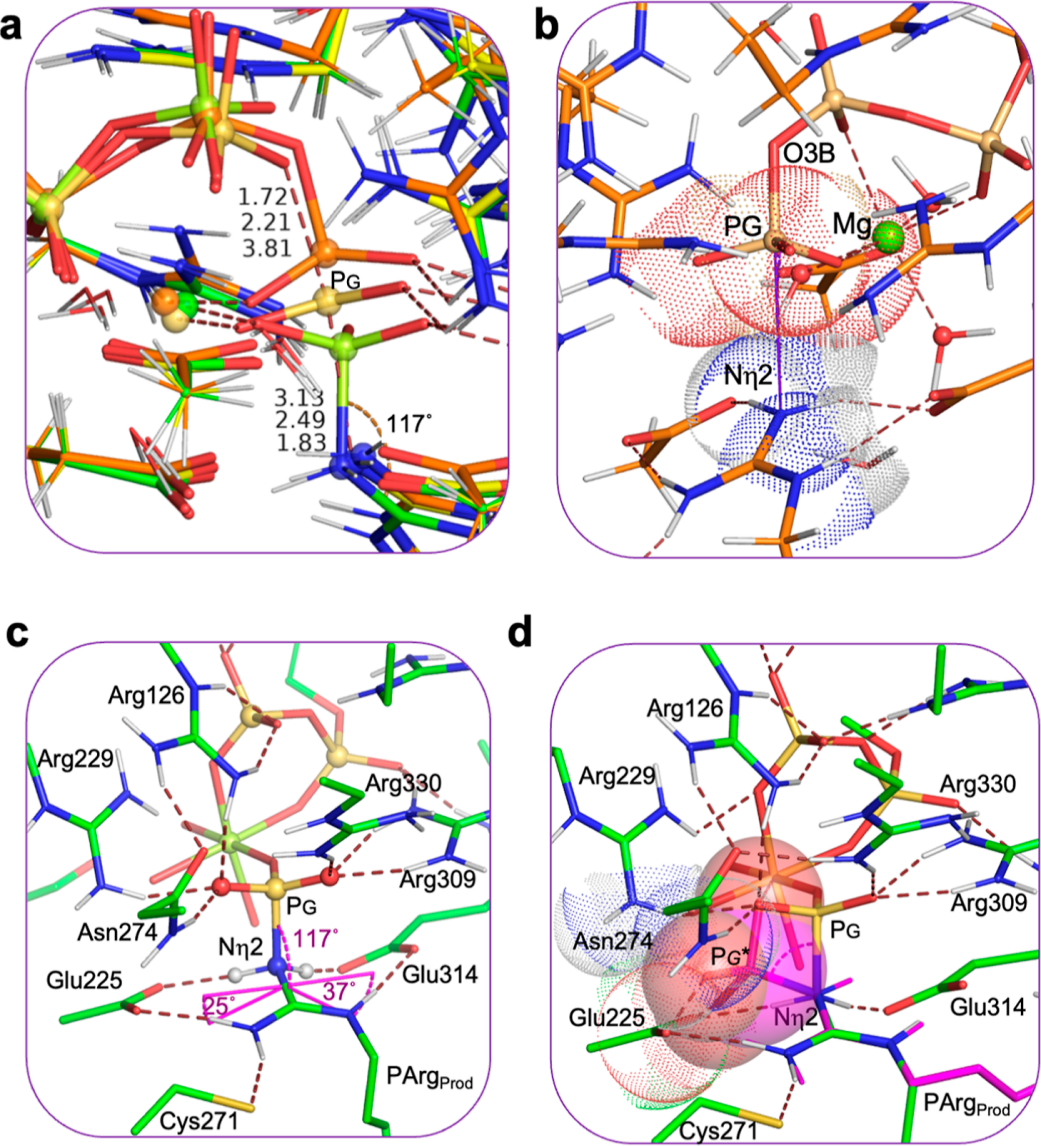
DFT-derived models for the reactant (orange sticks), TS (yellow sticks), and NPC (green sticks) cluster models. (a) These are aligned and focus on the movement of PG in the reaction (PA, PB, and PG color-coded to match). For the reactant, *r*_DA_ is 4.83 Å, shortening to 4.62 Å in the TS, and then expanding to 5.87 Å in the NPC complex, which has a P–N bond length of 1.83 Å. PG moves in-line, starting at 168°, increasing to 171° in the TS and closing at 161° in the first product complex, mainly facilitated by an “in–out” movement of PB. (b) Reactant model (orange sticks) with *r*_DA_ 4.83 Å bringing the atoms in the γ-phosphoryl group and the Nη2 amino group into van der Waals contact (dotted spheres). (c) NPC model (green sticks) formed by eigenvector development from the TS, with two protons (white spheres) on a fully tetrahedral Nη2. Its protons are out-of-plane by 25 and 37° dihedral angles to the guanidinium moiety. (d) van der Waals depiction of parts of (c) showing the steric clash between the phosphoryl group of a stable trigonal planar *N*-phosphoarginine, PArg_McsB_ (magenta sticks), selected from McsB (PDB: 6fh3) and aligned showing its in-plane phosphorus, (PG*, magenta sphere) and a single proton on Nη2 (magenta). The van der Waals surface of the trigonal planar PArg_McsB_ (PDB: 6fh3; orange and magenta spheres) clashes strongly with Arg229 and Asn274 (red and blue dots, van der Waals surfaces) as PG* is rotated 72° from the cavity position of PG.

This larger TS model was used to obtain models for AK·Mg·ATP·Arg_sub_ (“reactant”) and AK·Mg·ADP·PArg (“product”) complexes (Figure 4) by distorting the large TS model geometry along the eigenvector corresponding to the imaginary frequency eigenvalue with positive and negative unitary magnitudes for the product and reactant, respectively. The two geometries obtained from this process were optimized to obtain GS models for reactant and product, which both aligned well when overlaid with the original TS model (Figure 4a). Despite the geometry optimization of these models proceeding smoothly, the barrier (*E*(TS) – *E*(reactant) = 92–95 kJ mol^–1^) was significantly larger than that computed for the smaller model. In seeking to understand this discrepancy, we found that the computed energies of the TS and reactant models are highly dependent on the choice of dielectric for the implicit solvent (Table S4). Thus, it appears that implicit solvent models may not be suitable for obtaining accurate energies in this system. Indeed, the smaller system contains a large number of explicit water molecules (Figure S2). The highly charged and polarized nature of the active site presents challenges in selecting the appropriate dielectric constant; any calculation of accurate energetics may require the use of QM/MM methods,^34^ although it is unlikely that our structural conclusions will be unchanged in such studies as both models produce very similar TS geometries.

In the initial product complex, the two Nη2-guanidinium protons are hydrogen-bonded to nearby glutamate side chains, which we thus identify as a near-product complex (NPC) (Figures 4c, and S5). In the reactant complex, the PA–O3A–PB–O3B–PG atoms in ATP all coordinate the catalytic Mg^2+^ whose octahedral complex structure maps accurately on the equivalent Mg·ATP substructure in eight well-resolved X-ray structures (including PDB: 5fdx and 5gqm, at 1.30 and 1.68 Å, respectively). These structures contain the O3B–PG bond lengths of 1.60–1.65 Å, comparable to the distance of 1.72 Å present in the reactant cluster model. This small discrepancy can be attributed to the effect of the six hydrogen bonds between the protein and the three oxygens connected to PG in the computational model. A key feature of all these AK structures is that no water is found in the active site within 6 Å of PG other than the waters that complete the octahedral co-ordination of the catalytic magnesium ion, a feature widely observed for all enzymatic phosphoryl transfers.^35^

The reactant cluster model is best thought of as a near attack conformation (NAC)^36^ in which atoms of the γ-phosphoryl group are placed in van der Waals and H-bonding contact with the NH_2_ group of the guanidinium moiety, such that *r*_DA_ has a value of 4.83 Å (Figure 4b). The NPC model was also aligned with the computed TS by superimposing the 16 “fixed” carbon atoms in the two models (Figure 4c). The resulting overlay shows that O1G moves little as the reactants move to the NPC due to its tight coordination with the catalytic Mg^2+^, while O3G moves 1.0 Å with compensatory shifts for Arg279 and Asn274. Similarly, O2G moves 1.2 Å with a commensurate adjustment of Arg309 and Arg330. The kinase mechanism is thereby identified as an associative phosphoryl transfer that is mediated by a “swinging-arm” action of PB, with appropriate adjustment of residues to which it is H-bonded (Movie S1).

A remarkable feature of the NPC cluster model is that Nη2 of the *N*-phosphoarginine (PArg) exhibits an unusually long bond to PG (1.83 Å) and is tetrahedral because both protons are retained (Figure 4c). We know of no chemical precedent for such a structure: *N*-protonation of phosphoramidates, including *N*-phosphoarginine, leads to their spontaneous hydrolysis in water.^37^ Moreover, it follows that the guanidinium moiety of enzyme-bound PArg is initially formed as a *dication*. Our computed model suggests that this unique structure is stabilized by multiple H-bonds between the phosphoryl group and the side chains of Arg126, Arg229, Arg309, and Arg330, which sterically constrain the phosphorus atom to lie above the plane of the guanidinium group with an oop dihedral angle of 86° for Nε–Cζ–Nη2–PG.

**Figure 5:**
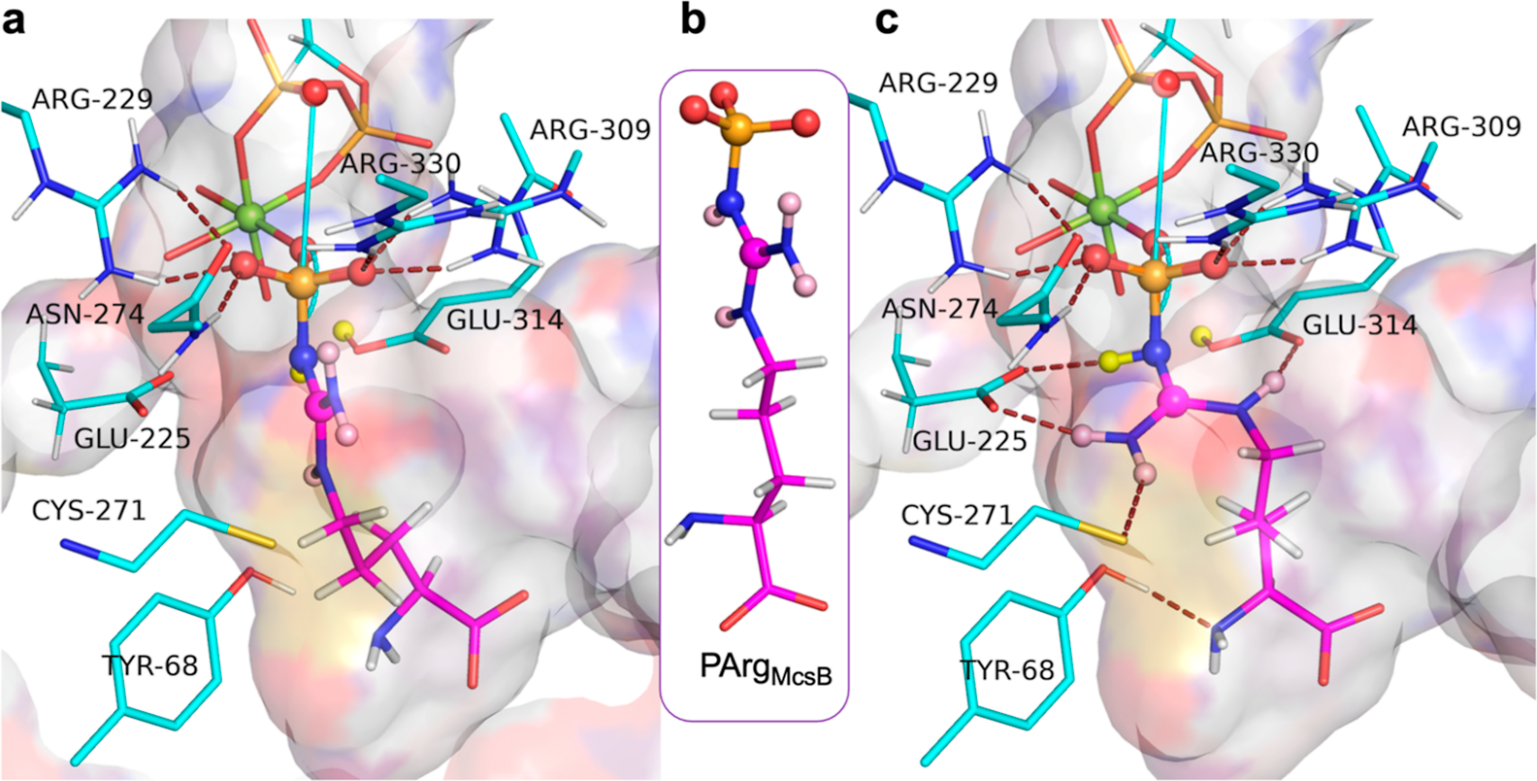
Accommodating *N*-phosphoarginine into the AK active site for the reverse reaction. (a) Product model structure (cf. Figure S4, cyan sticks) with PArg_McsB_ (magenta sticks) aligned on the phosphoramidate moiety by its CN_2_O_3_P atoms (0.17 Å rmsd) showing magnesium coordination and six H-bonds to Arg 229, 309, and 330 and to Asn274 (ruby dashes). The trigonal, planar phosphor-guanidinium geometry denies H-bonding to the proximate glutarate and cysteine residues. (b) *N*-Phosphoarginine from McsB crystal structure (PDB: 1fh3). (c) Same structure as in Figure 4a after ∼70° rotation of the Nη2–Cζ bond, which aligns the guanidinium group of PArg_McsB_ with that of the model product, enabling the formation of 5 regular H-bonds to the essential amino acids, Glu225, Glu314, and Cys271 (ruby dashes). The flexibility of the arginine C2–C5 carbon chain enables it to remain within the AK substrate channel that accepts ATP and arginine for the reaction (shadow surface).

To explore the origin of this pyramidalization, we shifted the proton on Nη2 toward OE2 of Glu314 by 0.1 Å and reoptimized the structure to yield a product complex with an uncharged Nη2. Quite remarkably, the two structures show only minor conformational differences. The H-bond from Nη2–Η···ΟΕ2 is 1.56 Å in the NPC structure and lengthens to 1.85 Å for the same proton in Nη2···H–ΟΕ2 in the product. The oop dihedral angle for PG moves to 74° and Nη2 shifts 0.2 Å; all other atom pairs align remarkably closely (Figure S6). Notably, the unusually long P–N bond in the NPC (1.83 Å) shortens to 1.75 Å in the product cluster model (Table S5). These values bracket the standard value of 1.78 Å for H_3_N^+^–PO_3_^–^.^38^ Our firm conclusion is that the enzyme-bound PArg has a tetrahedral Nη2 bond. Critically, such a structural reorganization cannot be delivered within the AK active site because a planar phosphoramidate group, as modeled by McsB,^39^ sterically clashes with the side chains of Glu225 and Asn274 (Figures 4d and 5). This shows that there is no possibility of binding a planar, trigonal PArg within the AK·Mg·ADP·PArg complex without major conformational reorganization. We anticipate that such protein conformational change must be linked to product ejection from the active site to complete the formation and release of the ultimate product, PArg.

AK enzymes are functionally reversible; therefore, how do these observations apply to the reverse reaction? The rapid formation of ATP needs to be initiated by protonation of Nη2 in the substrate PArg! We therefore envisage the reverse reaction to begin by Coulombic attraction of the trigonal planar, anionic phosphoramidate moiety in PArg into the strongly cationic cavity formed by Mg^2+^, Arg229, Arg309, and Arg330. These residues, along with Asn274, donate six H-bonds to the phosphoryl oxygen atoms (Figure 5b). However, they deny the donation of 5 H-bonds to Glu225, Glu314, and Cys271 from the guanidinium group while it maintains its trigonal planar character (Figure 5a). The rotation of the Cζ–Nη2 bond by ∼70° enables the formation of those H-bonds and positions the phosphoryl group perpendicular to the guanidinium plane (Figure 5c). This enforced conformational change promotes the basicity of Nη2 for protonation by Glu314 inside a cavity where there is no isolated water available to hydrolyze the protonated phosphoramidate. It is thereby positioned with PG, accurately aligned with O3B, setting up in-line nucleophilic attack on PArg, and facilitating phosphoryl transfer to generate ATP.

We note that this binding-activation process for PArg is completed only when the NTD closes on the arginine headpiece to enable the residues in Loop 62–68 to form the five H-bonds that are critical for arginine substrate recognition, as we built into our extended model for DFT analysis. It is, therefore, reasonable to suppose that PArg release/binding and NTD opening/closure are closely related activities that, between them, constitute the rate-determining step in AK catalysis of its forward and reverse reactions.

### Chemical Bonding in the Computational Cluster Models: Nitrate Anion Is Not a TSA

While a variety of strategies have been used to distinguish associative and dissociative transition states for phosphoryl transfer,^40,41^ quantum chemical methods, including QT-AIM,^42^ provide an unambiguous definition. We therefore computed the ED map at contours over a range of 0.80 to 0.10 e^–^ Å^–3^, for the Nη2–N_nt_–O3B plane of the AK-nitrate active site cluster model. These calculations show a clear discontinuity between Arg_sub_–Nη2 and N_nt_ (the nitrate nitrogen) and between O3B and N_nt_, across the entire range of ED values. Moreover, the ED contours for Nη2 and O3B show no oop polarization “bulge” in the direction of N_nt_ (Figure S7). These results support the analysis that there is no interaction between the nitrate anion and the donor and acceptor atoms in Mg·ADP and arginine, respectively. This finding is consistent with the absence of energetically accessible, unoccupied MOs on N_nt_ in the nitrate anion. As a result, nitrate is best identified as an adventitious “squatter” ligand that is only bound by phosphagen kinase active sites because of its three anionic oxygens and its geometrical relationship to an isolated metaphosphate anion (PO_3_^–^), an idealized component of an S_*N*_1P reaction mechanism (Table S6). These quantum chemistry-derived findings contradict multiple literature assertions that nitrate is a TSA,^12,43,44^ and challenge prior claims for dissociative mechanisms of phosphoryl transfer based on phosphagen kinase/nitrate complexes.

**Figure 6:**
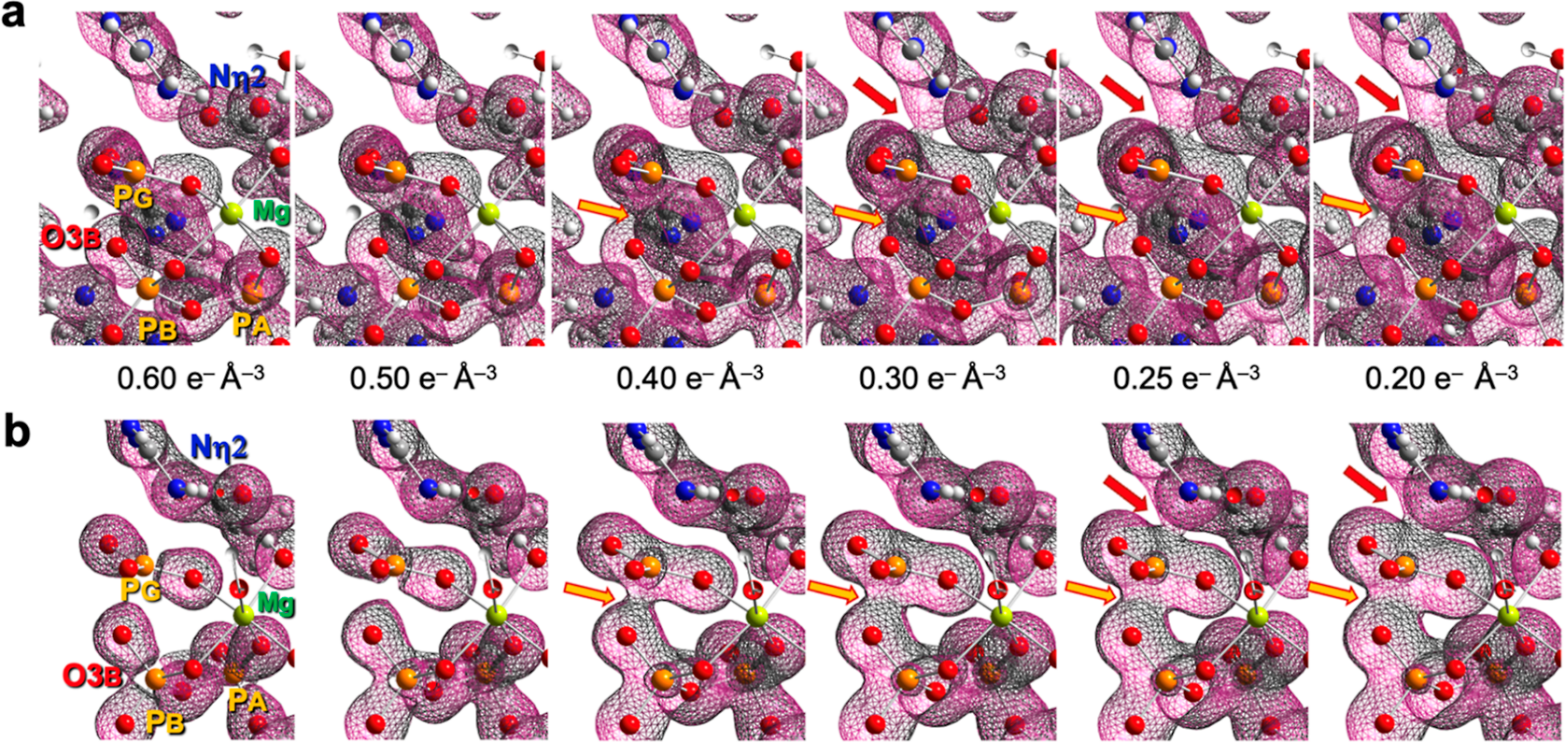
ED maps computed for the small AK·Mg·MeDP·PO_3_^–^·Arg_sub_ TS model. (a) Complete computed TS complex with Nη2, PG, and O3B in the *xy*-plane showing growth in the ED bulge on Nη2 from ≤0.50 e^–^/Å^3^ to the formation of the Nη2–PG ED at ≤0.30 e^–^/Å^3^ (red arrows). A similar merging of ED from O3B to PG is initiated at ≤0.40 e^–^/Å^3^ (gold arrows). (b) Equivalent ED calculated for a “non-enzymic” TS complex of 49 reactant atoms with locked coordinates after removal of all atoms located in catalytic residues. It shows no ED bulge on Nη2 from 0.60 to 0.40 e^–^/Å^3^ and linking ED to PG is seen only at ≤0.25 e^–^/Å^3^ (red arrows). A corresponding coalescence of ED from the O3B to the PG is seen at ≤0.40 e^–^/Å^3^ (gold arrows). Density maps and atoms/bonds are slabbed to +0.1 and +0.5 Å, respectively, along the *z*-axis. Only polar hydrogens are shown for clarity; (H, white; Mg, lime green; N, blue; O, red; P, orange).

Similar ED surfaces in the Nη2–PG–O3B plane of the small TS cluster model were mapped at contours from 0.6 to 0.2 e^–^ Å^–3^ (Figure 6a). Contiguous ED for the O3B–PG and PG–Nη2 bonds is observed at ED values of ≤0.40 and ≤0.30 e^–^ Å^–3^, respectively. These observations are consistent with phosphoryl transfer taking place via an early, unsymmetrical, associative TS. Moreover, at ED values >0.30 e^–^ Å^–3^, we observe a “bulge” of electrons on Nη2 (Figure 6a), directed toward PG. Although this electronic polarization on Nη2 is likely a consequence of pyramidalization in the TS involving the out-of-plane hydrogen-bonded carboxylates of Glu225 and Glu314, it might result (in part) from interactions involving oop Mg^2+^ and PG.

We therefore sought to understand the origin of the ED “bulge” on Nη2. These two cationic centers, PG and Mg_cat_, deliver a Coulombic attraction that is nearly 3-fold greater at the proximate Nη2 than at the more distant Nη1. The removal of all atoms in the catalytic amino acid moieties of the TS model removes the ED “bulge” on Nη2, with contiguous ED for the PG–Nη2 bond now seen at ED values ≤0.25 e^–^ Å^–3^ (Figure 6b). This result shows that the positive polarization seen at the nucleophilic nitrogen Nη2 in the complete TS cluster model is due predominantly to negative charge transfer from Glu225, Glu314, and Cys271, as previously recognized in quantum chemistry calculations on guanidinium acetate.^45^ A component of oop polarization, however, may be associated with pyramidalization of the reactive nitrogen in the guanidinium moiety.

## Conclusions

AK accelerates a fascinating chemical process: the delivery of an anionic phosphoryl group (PO_3_^–^) from ATP to a planar, trigonal nitrogen in a cationic guanidinium function. Our DFT calculations show that pyramidalization of the Nη2 acceptor and stereospecific polarization of charge onto the re-face of the guanidinium moiety yields a TS for in-line, associative phosphoryl transfer with a 4.66 Å separation of the donor oxygen and acceptor nitrogen atoms. Passing through this TS then gives a near-product complex, which is constrained by multiple H-bonds that hold the phosphoramidate group perpendicular to the guanidinium plane, thereby obstructing its relaxation to a stable, planar, trigonal, final product (Figure S8 and Table S5). The proton generated in the reaction (Figure 1a) is held within this complex prior to product release. We provisionally suggest that trigonalization and product release are concerted actions.

This DFT-derived proposal also explains two other experimental findings. First, the CK-catalyzed phosphorylation of creatine proceeds with stereochemical inversion of configuration at phosphorus, implying “an associative in-line transfer of the phosphoryl group between the bound substrates”.^46^ In light of the close evolutionary relationship between CK and AK, it seems reasonable to assume that both enzymes employ identical chemical mechanisms. Second, and perhaps more importantly, our model avoids the need to deprotonate the strongly basic guanidinium group (p*K*_a_ 13.8).^47^ As a consequence, there is no need to invoke the existence of an active site residue that can act as a general base catalyst. This prediction is again consistent with the experiment.^11^

Our analysis has wider relevance for understanding the mechanism of a broad range of post-translational modifications of proteins and nucleotides. For example, polarization and pyramidalization will likely facilitate methylation reactions catalyzed by protein arginine methyl transferases, PRMTs, in which types I, II, and III all have paired glutamates H-bonded to the substrate arginine.^48^ It is only a small step to suggest that is also the case for arginine glycosylation reactions that are well-established in many pathogenic bacteria.^49^ Last, but by no means least, methylation of trigonal, planar nitrogens to give *N*-6 adenosine and *N*-4 cytidine nucleobases in DNA and RNA, respectively, is a feature of epigenetic activation.^50−53^

## Data Availability

All data supporting this study is provided as Supporting Information accompanying this paper.

## Supporting Information Available

Details of computational methods, models and energetics, computed structures for selected cluster models comparison of the computed NPC and product cluster models, electron density surface map, examples of DFT analyses for selected kinases, thermodynamic data for arginine kinase, summary of the hydrogen bonds in the AK TS and AK-nitrate cluster models, summary of enlarged TS cluster model energetics, computed bond lengths and angles for the AK-nitrate, small TS and large TS cluster models, and coordinates for all cluster models (PDF)
Motion of P_B_ along the imaginary vibrational mode in the TS (MP4)

## Footnotes

1 Here, and throughout the paper, names for atoms in the phosphate groups are consistent with IUPAC 2016 recommendations.^31^
