# Supplementary material for: Arginine Kinase Activates Arginine for Phosphorylation by Pyramidalization and Polarization
Source: ACS Catal. 2024 Apr 16;14(9):6650–8. doi: 10.1021/acscatal.4c00380 (PMC11075012; doi:10.1021/acscatal.4c00380)
Supplement: Supplementary file 1 — cs4c00380_si_001.pdf [file cs4c00380_si_001.pdf]

Arginine Kinase Activates Arginine for Phosphorylation by Pyramidalization and Polarization

Fabio Falcioni *Department of Chemistry, University of Manchester, Manchester M13 9PL, UK*; orcid.org/0000-0003-4041-6695.

E-mail: fabio.falcioni@manchester.ac.uk

Robert W. Molt, Jr. *Department of Biochemistry and Molecular Biology, Indiana University School of Medicine, Indianapolis, IN 46202, USA*; orcid.org/0000-0002-2765-8713.

E-mail: r.molt.chemical.physics@gmail.com

Present address: *ENSCO Inc., Melbourne, FL 32940, USA*

Yi Jin *Manchester Institute of Biotechnology, The University of Manchester, Manchester M1 7DN, UK*; orcid.org/0000-0002-6927-4371.

E-mail: yi.jin@manchester.ac.uk

Jon P. Waltho *Manchester Institute of Biotechnology and Department of Chemistry, University of Manchester, Manchester M1 7DN, UK; School of Biosciences, University of Sheffield, Sheffield S10 2TN, UK*; orcid.org/0000-0002-7402-5492.

E-mail: j.waltho@manchester.ac.uk

Corresponding Authors

Sam Hay *Manchester Institute of Biotechnology and Department of Chemistry, University of Manchester, Manchester M1 7DN, UK;* orcid.org/0000-0003-3274-0938.

E-mail: Sam.Hay@manchester.ac.uk

Nigel G. J. Richards – *School of Chemistry, Cardiff University, Park Place, Cardiff CF10 3AT, UK*; *Foundation for Applied Molecular Evolution, Alachua, FL 32615, USA*; orcid.org/0000-0002-0375-0881.

E-mail: nrichards@ffame.org

G. Michael Blackburn – *Leverhulme Emeritus Research Fellow*, *School of Biosciences, University of Sheffield, Sheffield S10 2TN, UK*; orcid.org/0000-0002-3941-0459.

Email: g.m.blackburn@sheffield.ac.uk

Supporting Information

Contents

1. Computational Methods p. 3
2. Supplementary Figures p. 7
3. Movie p. 15
4. Tables p. 16
5. References p. 21
6. Coordinate data for the computed model structures p. 24
    a. Small AK-nitrate cluster model p. 24
    b. Small TS cluster model p. 27
    c. IRC-derived reactant cluster model p. 31
    d. Large TS cluster model p. 35
    e. Large Reactant cluster model p. 42
    f. Large NPC cluster model p. 49
    g. Large Product cluster model p. 56

1. Computational Methods

a. Small Cluster Model of the AK·Mg·ADP·NO_3^-·Arg_{sub} ("AK-Nitrate") Complex.

An initial active-site cluster model was built from the X-ray crystal structure of the AK·Mg·ADP·NO_3^-·Arg_{sub} complex (PDB: 1m15).[1] This cluster model (229 total atoms and a total charge of +1 in a singlet state) contained the nitrate anion, Mg^{2+} with its three coordinated waters (w1296, w1297 and w1298), methyl diphosphate (MeDP) representing truncated ADP, the substrate arginine (Arg_{sub}), residues Arg124, Arg126, Glu224, Glu225, Arg229, Cys271, Asn274, Arg280, Arg309, Gly313, Glu314 and Arg330, and the water (w1220) seen in the X-ray crystal structure because it forms a core set of H-bonds to O3G, Arg330, and Thr273. Five other crystallographic waters, w1210, w1212, w1216, w1219, and w1223, which contribute 12 hydrogen bonds in the catalytic region, were included in the model. All arginine and glutamate side chains were modelled as their cationic and anionic forms, respectively. Protein residues and Arg_{sub} were truncated with carbons at the truncations being fixed at their crystallographic locations. Similarly, CA of the substrate arginine was treated as a fixed carbon. Thus, the model contained components from 13 protein residues with 14 peripheral carbons being constrained to their crystallographic coordinates. In addition, four crystallographic water molecules (w1214, w1215, w1220, w1282) were included in this initial cluster model, together with two waters that were positioned to form hydrogen bonds that would minimize artefactual charge density effects at the boundary of the model (Figure S2).[2,3] We anticipated that these latter waters would not significantly impact the geometric features at the center of the cluster.

As in our previous studies of phosphoryl transfer in GTPases,[4,5] DFT calculations on the model were performed using the M06-2X functional,[6] which provides a good description of hydrogen bonds and dispersion interactions,[7] and a cc-pVDZ basis.[8] Diffuse functions (aug-cc-PVDZ)[9] were assigned to atoms possessing a formal negative charge (cysteine and glutamate side chains) and in MeDP. In order to describe polarizability, we assigned a cc-pVTZ basis to the Mg^{2+} ion and all phosphorus atoms. Finally, an aug-cc-pVTZ basis[8,9] was assigned to the three oxygen substituents of the nitrate anion, O3B of MeDP (nomenclature follows ref. 10) and the nucleophilic nitrogen (Nη2) in Arg_{sub}. SCF wavefunctions were considered converged when elements in the density matrix changed by less than 10^{-6} between iterations. Geometry optimizations required a total RMS force of less than 10^{-4} Hartrees/Bohr (individual force components could not have a value that exceeded 3.3×10^{-4} Hartrees/Bohr). The Lebedev integration grid had 130 radial points and 770 solid angle integration points. Harmonic vibrational analysis confirmed the validity of all stationary states on the potential energy surface; analytic gradients and Hessians,[11-13] together with spherical *d* functions, were used in all calculations. These computations were performed using Gaussian09,[14] with electron densities being visualized in Gaussview.[15]

b. Small Cluster Model of the TS for AK-Catalyzed Phosphoryl Transfer.

The initial AK·Mg·ADP·PO_3^-·Arg_{sub} cluster model was built from the optimized model of the AK·Mg·ADP·NO_3^-·Arg_{sub} complex by replacing the nitrate nitrogen by phosphorus. All basis assignments were identical to those used above, and the phosphorus atom in the phosphoryl group undergoing transfer was assigned as cc-pVTZ. Identical constraints on the peripheral carbon atoms to those used for the AK-nitrate model were present. A standard transition state (TS) search was performed using the Schlegel algorithm,[16] as implemented in Gaussian09. The resulting TS cluster model (Figure S2) had a single imaginary, harmonic vibrational mode (233*i* cm^{-1}) for motion along the reaction coordinate. Small imaginary frequencies associated with librations of methyl groups containing constrained carbon atoms were ignored.

We also attempted to locate a dissociative TS by setting the initial distances for the reacting atoms at the values seen in the AK-nitrate cluster model. Despite computing force constants and confirming a ground-state electronic wavefunction at each search step, the system converged to an associative TS.

c. IRC Calculations Starting from the Small TS Cluster Model.

This small TS cluster model was used as the starting point in IRC calculations to locate the associated AK·Mg·ATP·Arg_{sub} (reactant) cluster model. All basis assignments and atom constraints were identical to those used to obtain the AK-nitrate and TS models. The search for the reactant cluster model was accomplished in 27 steps (0.1 Bohr/√AMU) (see below) using Schlegel's method[17] as implemented in Gaussian16.[18] once again, spherical *d* functions were used in these calculations. We estimated the ΔH value for the barrier by taking the difference in energies of the small reactant and TS cluster models (Figure S2).

d. Large Cluster Model of the TS for AK-Catalyzed Phosphoryl Transfer.

An initial AK·Mg·ADP·NO_3^-·Arg_{sub} model was built based on the X-ray crystal structure 1m15. This cluster model (275 total atoms) contained most of the atoms in the small QC cluster model (see above) plus truncated forms of residues Ser63, Gly64, Val65, Gly66 and Tyr68. Only crystallographic waters were included. Protein residues and Arg_{sub} were truncated with carbons at the truncation points being fixed at their crystallographic positions. Given the relatively large number of atoms, this model was partially optimized using the M06-2X functional with a cc-pVDZ basis set being assigned to all atoms.

The initial AK·Mg·ADP·PO_3^-·Arg_{sub} cluster model (275 atoms and a total charge of -1 in a singlet state) was built from the partially optimized model of the AK·Mg·ADP·NO_3^-·Arg_{sub} complex by replacing the nitrate nitrogen by phosphorus. Before performing the transition state search, the system was relaxed with distance constraints being placed on the O3B-P and P-N2 bonds. Subsequent DFT calculations on this large TS model used the M06-2X functional.[6] A cc-pVDZ basis[7,8] was assigned to all atoms in the energy minimizations prior to obtaining the optimized large TS cluster model. For the TS search, the initial Hessian was obtained at the cc-pVDZ level of theory. Diffuse functions (aug-cc-PVDZ)[8] were then assigned to atoms possessing a formal negative charge (cysteine and glutamate side chains) and in MeDP. In order to describe polarizability, we assigned a cc-pVTZ basis to the Mg^{2+} ion and all phosphorus atoms. Finally, an aug-cc-pVTZ basis[7,8] was assigned to the three oxygen substituents of the phosphoryl group undergoing transfer, O3B of MeDP and the nucleophilic nitrogen (Nη2) in Arg_{sub}. The resulting large TS model was embedded in a conductor polarizable continuum model (C-PCM),[19] setting $\varepsilon = 4$, throughout all the calculations. All calculations to obtain ground state and transition state models used the Berny algorithm,[20] as implemented in Gaussian09. SCF wavefunctions were considered converged when elements in the density matrix changed by less than 10^{-6} between iterations. Geometry optimizations required a total RMS force of less than 10^{-4} Hartrees/Bohr (individual force components could not have a value that exceeded 3.3 x 10^{-4} Hartrees/Bohr). The Lebedev integration grid had 99 radial points and 590 solid angle integration points. Analytic gradients and Hessians were used in all calculations. The resulting TS cluster model had a single imaginary, harmonic vibrational mode (259*i* cm^{-1}) for motion along the reaction coordinate. Small imaginary frequencies associated with librations of methyl groups containing constrained carbon atoms were ignored.

e. Large Cluster Models of the Reactant and Products Complexes.

Large cluster models for the reactant and product complexes were obtained by using the imaginary frequency eigenvalues and eigenvectors to distort the geometry of the TS model with a positive unitary magnitude for the product and a negative unitary magnitude for the reactant. The corresponding geometries obtained from these distortions were optimized to yield ground state structures. The converged structure for the product search obtained the substrate arginine with the acceptor nitrogen in a tetrahedral doubly protonated state, H-bonded to Glu225. It was thus deemed to be a Near-Product-Complex (NPC). Given that the NPC was a local minimum, the proton on the phosphorylated guanidinium group that forms an H-bond with Glu255 was shifted 0.1 Å in the model and the resulting new product complex was re-optimized to obtain a model in which the phosphorylated guanidinium group had become deprotonated. This second

cluster model of the product complex was 3.9 kcal mol^{-1} more stable than the NPC (Table S4). Only minimal additional structural rearrangement of the model was observed and, given that the barrier for this proton transfer is expected to be small, the NPC can be considered a quasi-stable intermediate along the path from TS to product. Single point potential energies for the ground state structures were also computed for these models using with a better SCF convergence (residual of the density matrix of 10^{-8}) and Lebedev integration grid (175 radial and 974 solid angle points for first-row atoms and 250 radial and 974 solid angle points for atoms in the second and later rows).

2. Supplementary Figures

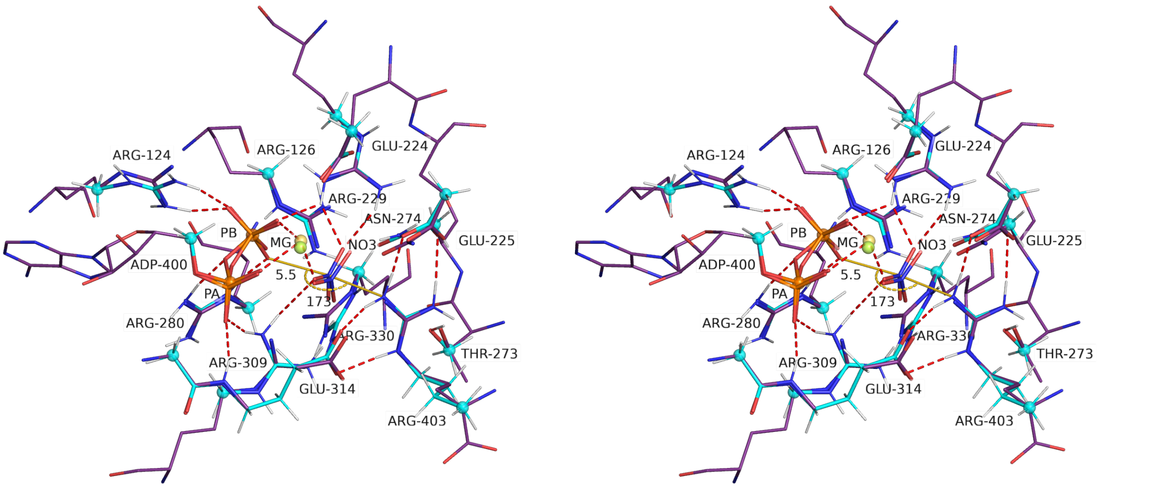

Supplementary Figure S1. Computed structure for the small $AK\cdot Mg\cdot MeDP\cdot NO_3^{-}\cdot Arg_{sub}$ cluster model (cyan sticks) aligned by 13 “locked” carbons (cyan spheres) on the 1.2 Å crystal structure (PDB: 1m15; purple). Hydrogens involved in 4 primary H-bonds to substrate arginine (Arg403) and 13 to the oxygens of PA, PB, and NO_3^{-} (red dashes). The catalytic magnesium (MG, lemon sphere) is shown coordinated to O1A, O1B, and nitrate (dashed lines). Donor-acceptor relationship for O3B and Arg_{sub}-N2 (η2) shown in yellow with in-line angle 173˚ and $\boldsymbol{r}_{DA}$ 5.5 Å (polar hydrogens, silver only for computed 1m15; magnesium, lime green; nitrogen, blue; oxygen, red; phosphorus, orange; uncoordinated waters omitted for clarity; data presented in PyMOL; 3-D presentation for wall-eyed viewing).

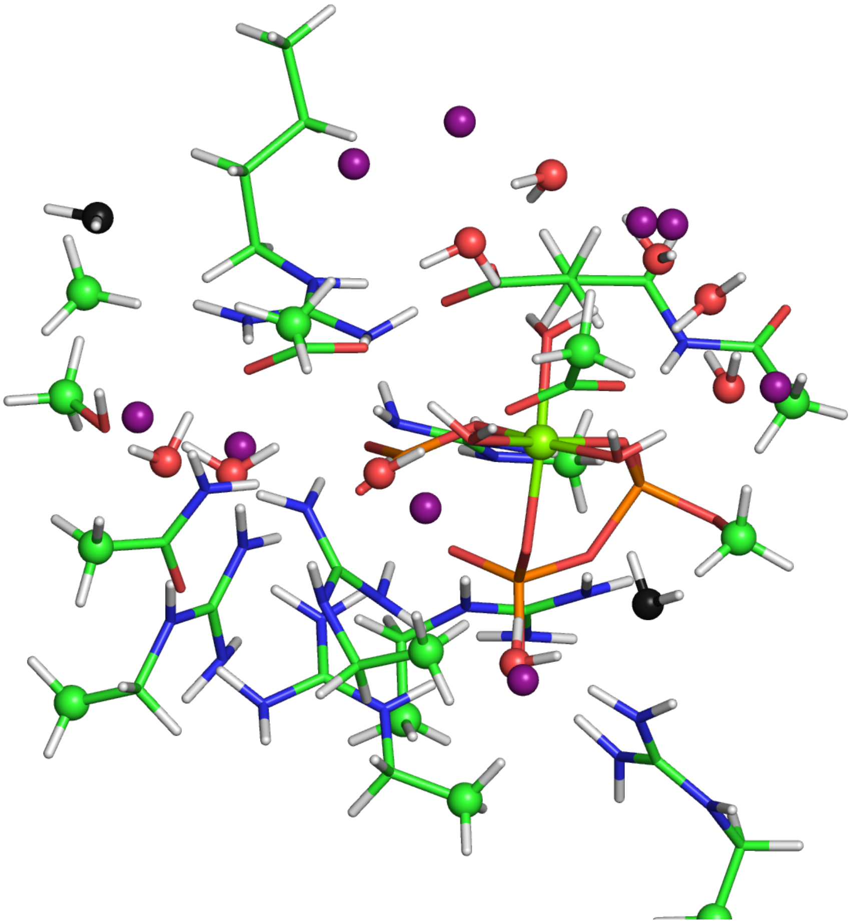

Supplementary Figure S2. Computed structure for the small TS cluster model showing the 13 “locked” carbons (green spheres) and the 14 water molecules derived from crystallographic waters (red spheres) and two additional waters (black spheres) positioned to hydrogen bond to adjacent polar atoms in the cluster, thereby minimizing artefactual charge density effects at the boundary. The original positions of the crystallographic waters (PDB: 1m15; w296, w297, w298, w1210, w1212, w1214, w1215, w1216, w1219, w1220, w1223, w1282) prior to geometry optimization are indicated by purple spheres (hydrogens, white; carbon, green; magnesium, lime green; nitrogen, blue; oxygen, red; phosphorus, orange; data presented in PyMOL).

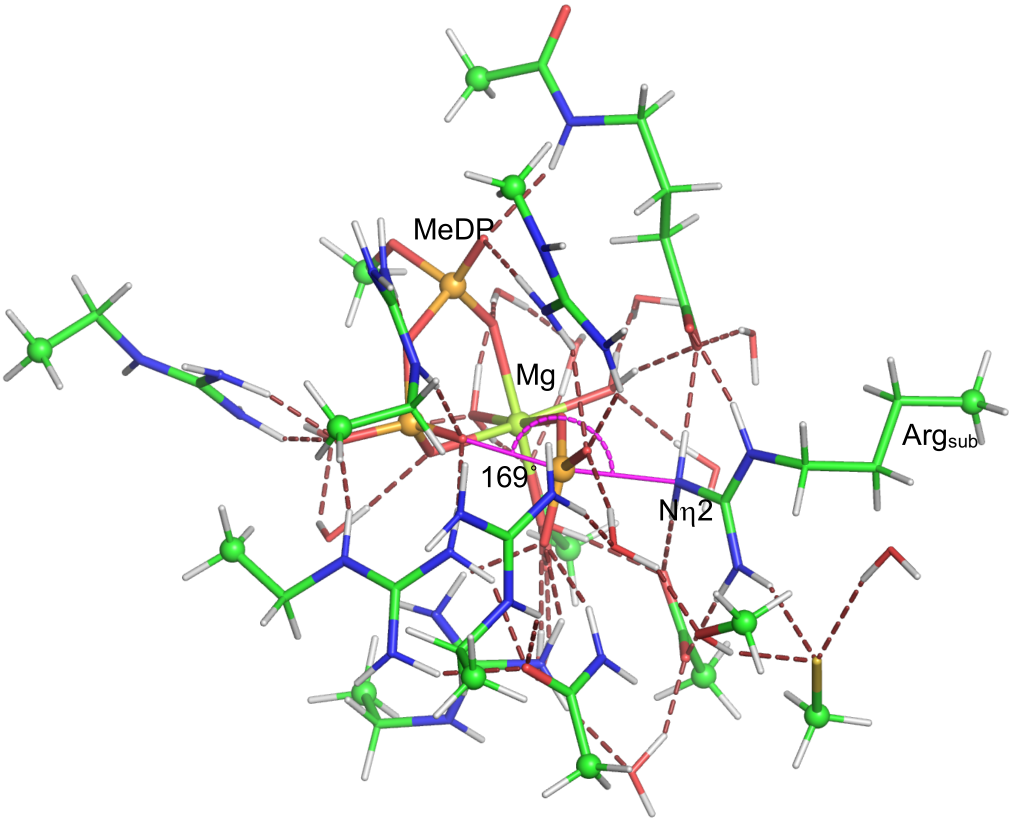

Supplementary Figure S3. The optimized structure for the small cluster model of the AK·Mg·ATP·Arg$_{sub}$ (reactant) complex obtained from the IRC calculations (green sticks). The donor-acceptor distance ($\boldsymbol{r}_{DA}$ is 4.56 Å and the in-line angle is 169˚ (magenta arc). The 14 'fixed' carbons are shown as spheres including that for Arg$_{sub}$ for this 'constrained' model. Only polar hydrogens are shown (60% width). 46 H-bonds are shown for the computed TS structure, focused on coordination of the enzyme to the substrate arginine and to the Mg.ATP complex, of which 10 are contributed by uncoordinated waters (phosphorus, orange spheres; magnesium, lemon sphere; Structure rendered in PyMOL).

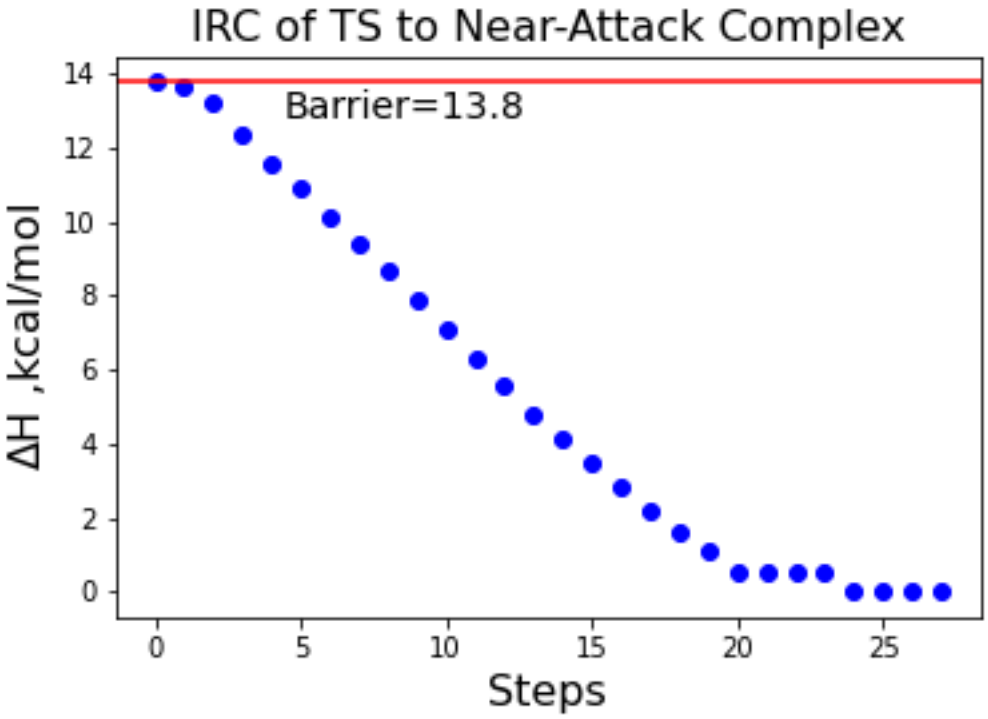

Supplementary Figure S4. Plot of ΔH (kcal/mol) along the intrinsic reaction coordinate leading from the small TS cluster model (step 0) to a small reactant (ATP + Arg) cluster model (step 27).

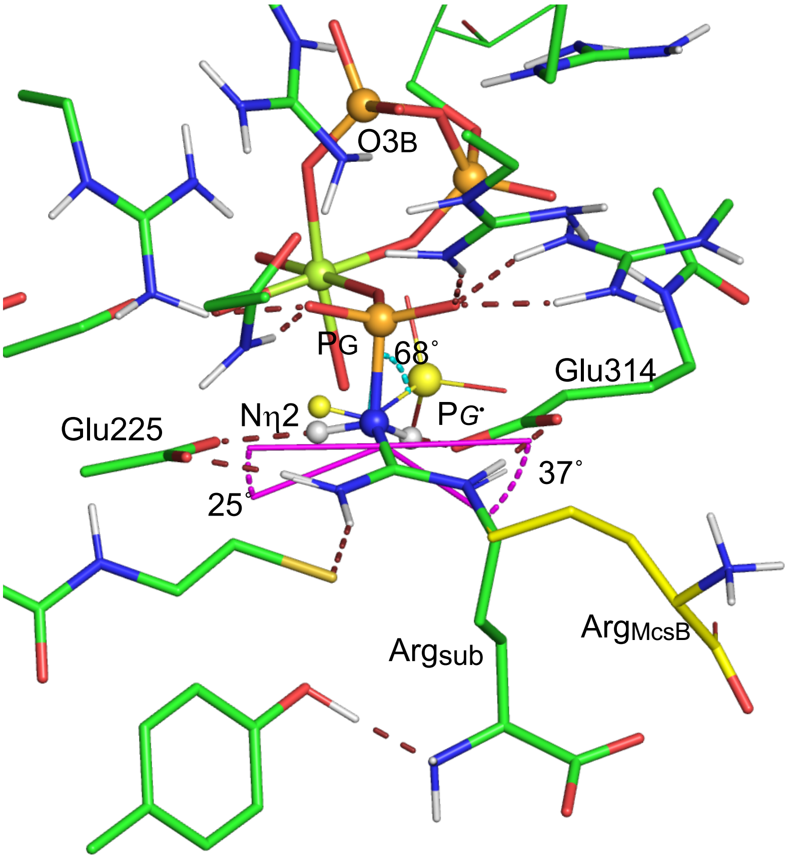

Supplementary Figure S5. Computed NPC cluster model for AK (green sticks). This is aligned with a planar (*S*)-phosphoarginine (yellow sticks) (PDB: 5hbn). The initial NPC product of the AK reaction depicts P_G (orange sphere) from the AK TS fully bonded to Nη2 of the substrate arginine ($\boldsymbol{r}_{PN}$ 1.65 Å), separated from $O3_B$, and with 2 intact hydrogens (white spheres) on the tetrahedral, cationic Nη2 of Arg_{sub}. Proton transfer of the (*Z*)-hydrogen (white sphere) of this ammonium ion to Glu314 will convert the NPC into the Product structure, which remains tetrahedral (see Figure S6). The terminal conformation of an AK-bound trigonal, planar PArg is illustrated by mapping onto the NPC the PArg structure from a *B. subtilis* McsB arginine kinase (mapping made using the paired guanidinium atoms in PyMOL). The final relaxation of the NPC into the plane of the guanidinium phosphate product calls for a rotation of P_G-Nη2-P_{G^*} of 8° (cyan arc) and severance of its coordination to Mg_{cat} and Arg229 which is impeded by vdW clashes (see Figure 4d) (hydrogen, white; magnesium, lemon; nitrogen, blue; oxygen, red; phosphorus, orange).

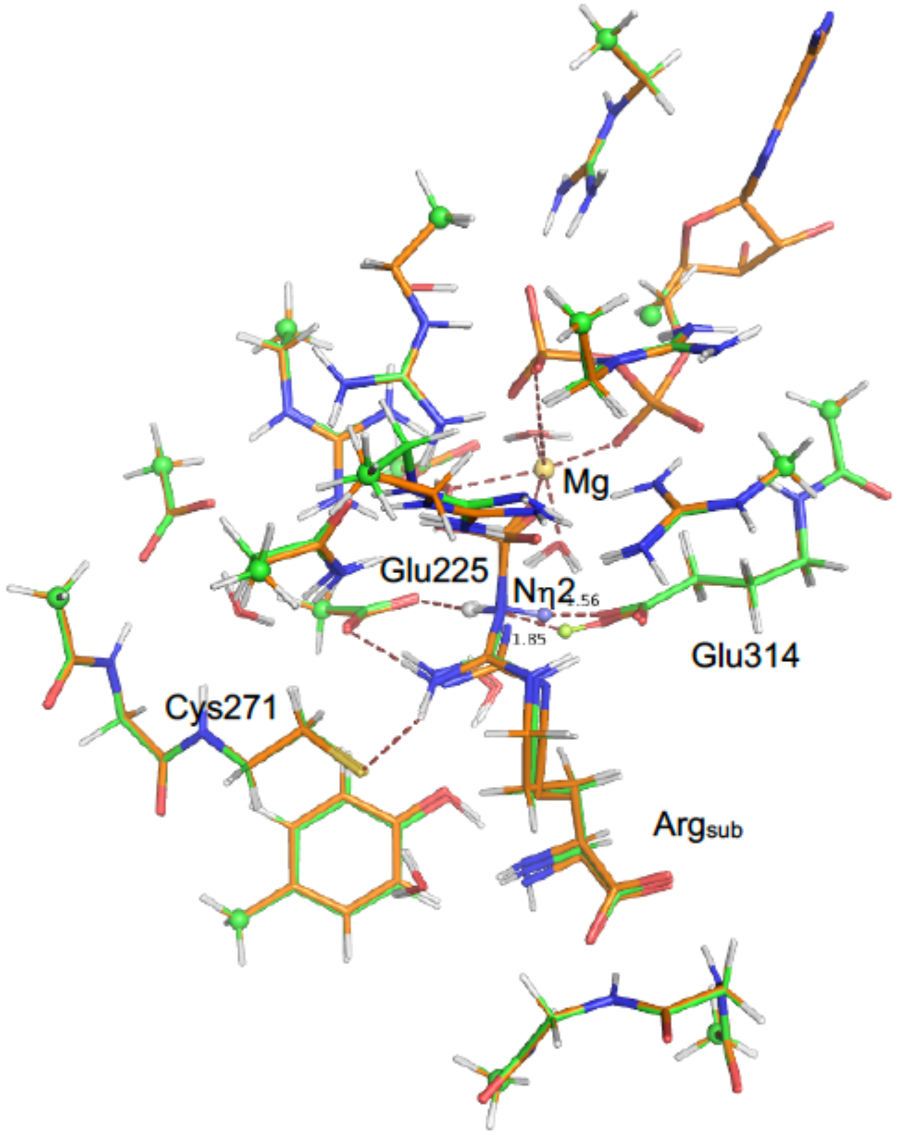

Supplementary Figure S6. Comparison of the computed Near Product Complex (NPC) and Product cluster models. The two structures were each superposed on 1m15 using 16 fixed carbons (green spheres) and show the NPG (green sticks) with a 1.56 Å H-bond from Nη2 to Glu314 for the NPC. After a 0.1 Å shift of that proton (light blue sphere) and re-computation, the Product structure (orange sticks) has that mobile proton (light green sphere) in a 1.85 Å H-bond from Glu314 to Nη2. The P-N bond length in the NPC (1.83 Å) has shortened to 1.75 Å in the Product structure, while the dihedral angle Nε-Cζ-Nη2-PG has diminished from 86.2° to 74.0°. H-bonds (dashes) are also shown for the guanidinium moiety to Glu215 and Cys271.

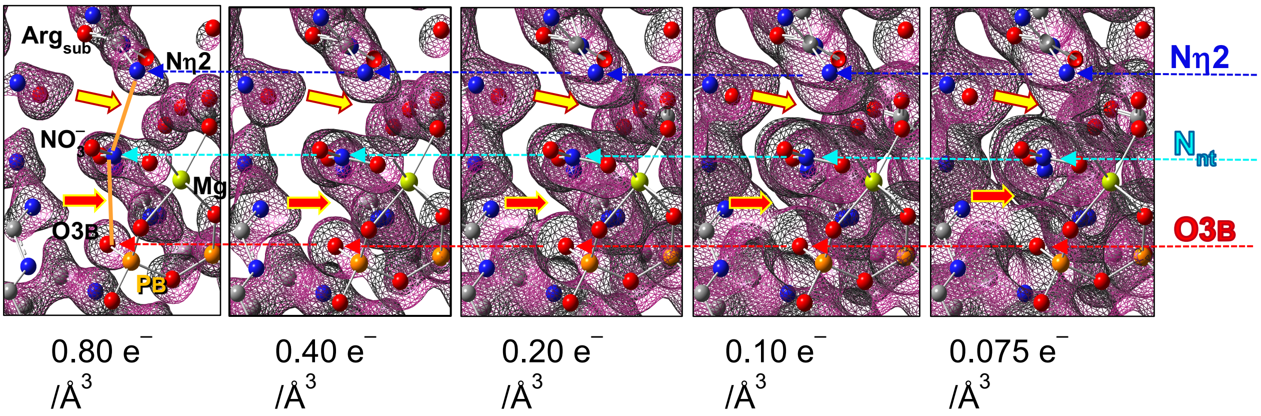

Supplementary Figure S7. Electron density surface map from 0.80 to 0.075 $e^-/Å^3$ computed for the ternary AK nitrate complex (AK·ADP·NO_3^-·Arg) (PDB: 1m15) with Nη2, PG, and O3B in the X-Y plane, (density slabbed to +0.2 Å and atoms slabbed to 0.7 Å in front of the X-Y plane). Positions of Nη2, N_{nt} and O3B highlighted by dashed horizontal lines. Void in ED between envelopes for N_{nt} with Nη2, and for N_{nt} with O3B indicated (gold and red arrows) (all hydrogens present in the computed ED but omitted for clarity). The 'in-line' angle of 161° is indicated by orange lines in the left-hand ED map (0.80 $e^-/Å^3$).

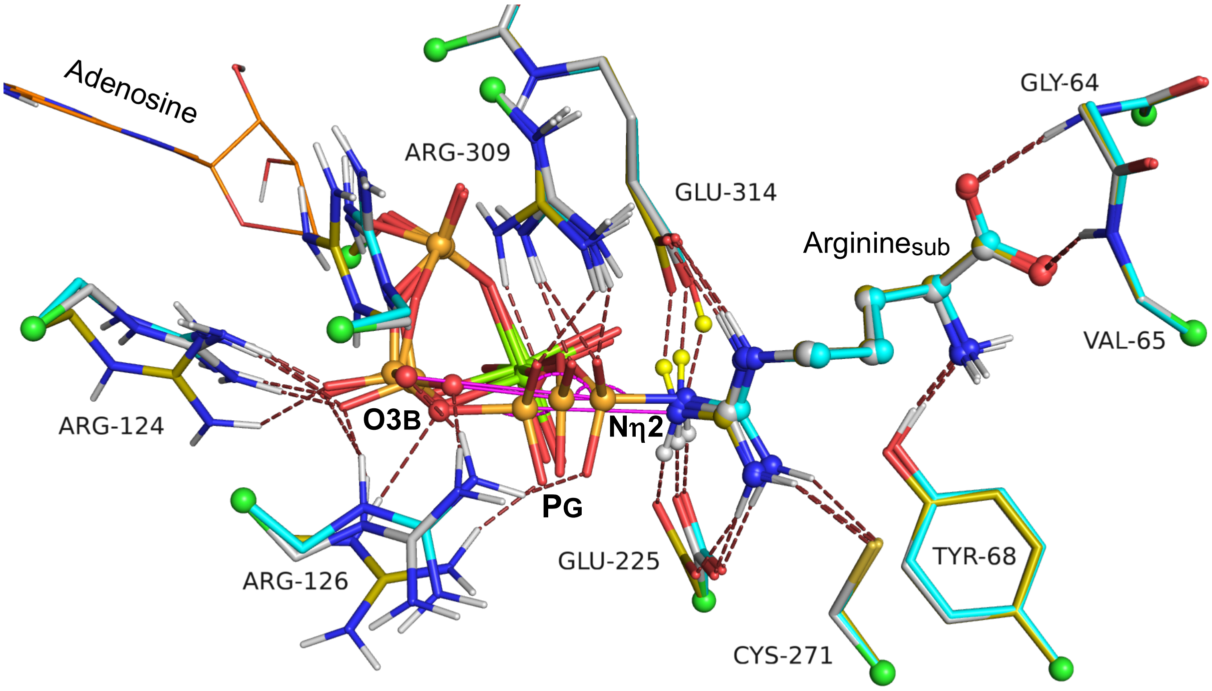

Supplementary Figure S8. H-Bonds controlling AK progression from Reactant to TS to Product. The model Reactant (olive sticks), TS (silver sticks) and Product (cyan sticks) structures are aligned pairwise with 1m15 using fixed carbons (11 shown). The reaction trajectory is from ATP (left, thin orange lines) to PArg (right, cyan spheres) as indicated by the in-line angles for each of the three stages (magenta lines, center). H-Bonds (ruby dashes) show coordination of the arginine headpiece to (i) residues in Loop 62-68 (right), (ii) the guanidinium moiety from Glu225, Cys271, and Glu314 (center), and (iii) the three phosphoryl groups from Arg124, Arg126, Asn274, and Arg309 (left and left-center). Major features are (a) the linear progression of PG as it leaves O3B and joins Nη2 (center), (b) the movement of O3B first towards Nη2 to form the TS then secondly its recoil to release PG, and (c) the track of the mobile (*Z*)-proton on arginine-Nη2 (yellow spheres) as its out-of-plane angle increases from reactant to TS and then migrates to Glu314/OE2 in the Product structure (Glu224 and Gln274 have been omitted for clarity).

4.

Movie S1

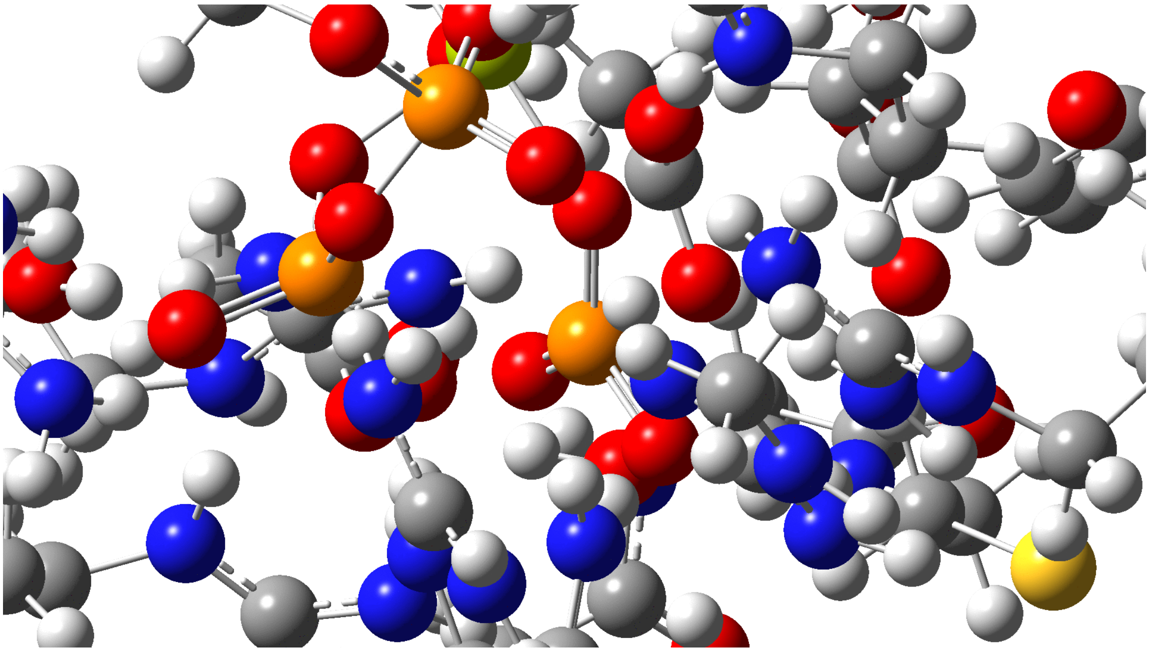

PA, PB, are orange spheres upper left and left, Mg is top rear (lime green). PG is center and oscillating with O3B (red sphere) left center and oscillating. Substrate arginine is right center with Nη2 (blue sphere) oscillating. Its two hydrogens (small white spheres) are above it and near motionless, being closely H-bonded to Glu225 and Glu314 (OE2, red spheres above, partly hidden). The oscillatory motion of PG is clearly linked reciprocally to that of Nη2 which is strongly pyramidalized as it closes on PG, and to O3B whose motion is linked to rotation of the O3A–PB bond. (Movie of the motion along the reaction coordinate taken from the large TS cluster model).

5. Supplementary Tables

Supplementary Table S1. Examples of DFT Analyses for Selected Kinases.

Protein	PDF Resolution / Å	DFT Method/ (Year)(ref)	Ligand or TSA	Number Heavy Atoms	Total Atoms	Number Amino Acids	Number H-bonds	Mg_A coordn	Mg_B coordn	r_{DA} / Å	$r_{D\text{-}PG}$ / Å	$r_{PG\text{-}A}$ / Å	In-line Angle°	Conclusion argument	Remarks
cAPK	1L3R 2.0 Å	B3LYP (2007)[15]	ADP.AF3	24	31	4 + Ser	6	O1B-O1G	O1A-O2G	≤ 4.64	2.41	2.23	n.g.	94%Dissociative Pauling/Mildvan	H^+ tx late in TS
		B3LYP/6-31+G(d) (2014)[16]	ADP.AF3	36	48	4 + S^{17}	4	O1B-O1G	O1A-O2G	≤ 4.11	1.81	2.30	n.g.	Associative mechanism with CBH proven	H^+ tx to O3G concerted
		B3LYP/ 6-31G(d′) (2013)[17]	ADP.MGF (1L3R re-refined)	–	339 *(102)*‡	10 + S^{21}	12	O1B-O1G	O1A-O2G	4.38	2.41	1.98	170.3°	Associative with CBH proven	H^+ tx to Asp^{166} concerted
InsRecep Kinase	1IR3 1.9 Å	B3LYP (2015)[18]	ANP	36 (48)	76	3 + Y_{sub}	5 (?)	O1B-O1G	O1A-O2B	4.2 ± 0.2	2.2	2.2	n.g.	Allosteric control by Pylation speeds H^+ tx	Pre TSA H^+ tx to D^{1132}
DgkA	3ZE5 3.1 Å	B3LYP/ 6-31G(d) (2015)[19]	ATP dock trimer	79		10 + DAG	10 several dubious	O1B-O1G	O1A-O3B (dock)!!	≤ 4.55	2.34	2.21	n.g.	88% Dissociative Pauling/Mildvan	H^+ tx late in TS
IPK	3LKK 2.0 Å	B97d / 6-31g(d) (2017)[20]	ATP No Mg	52	104	8 + IPP	5 in QM	O1A-O1B-O1[illegible]	–	≤ 4.02	2.00	2.02	n.g.	66%Dissociative Pauling/Mildvan	
GALK	1WUU 2.5 Å	B3LYP/ (AMBER) (2013)[21]	ANP + Galactose	49	68	5 + Gal	Not clear	O1A-O1G	–	≤ 4.06	2.00	2.06	n.g.	65% Dissociative Pauling/Mildvan	Direct H+ tx. No GABC w. Asp^{186}

‡ 227 atoms were constrained (*i.e.* locked), leaving 102 unconstrained.
CBH: charge balance hypothesis.[22]

Supplementary Table S2. Thermodynamic Data for Arginine Kinase.

AK Species	Assay	Solvent / pH	Temp / K	$\Delta H^{\ddagger}$ / kJ/mol	$\Delta G^{\ddagger}$ / kJ/mol	$\Delta S^{\ddagger}$ / J/K
Limnus polyphemus [22]	ADP formation	Water 8	297.3 – 302.1	33.9	65.7	-0.105
Homarus vulgaris[23]	pArg formation	1) Water 8.5<br>2) 40% glycol	293<br>293	52<br>52	61<br>65	-25<br>-42
Neocaridina denticulate[23] *AK2*	ADP formation	Water 8	288 – 308	48.1	59.6	-38.6
Corbicula fluminea[25]	ADP formation	Water 7.9	290.5 - 298	41.9	66.6	-83
Carcinus maenas[26]	*^{31}P NMR ATP formation*	*Saline (in vivo) ca. 8*	*296*	*n.a.*	*126*	*n.a.*

Reactions assays in the forward direction, PArg and ADP formation, with exception of Entry 5 where ATP was monitored by ^{31}P NMR.
For Entry 1, we used raw data to calculate the thermodynamic parameters shown.

Supplementary Table S3. Data for the 30 Primary H-bonds Computed for the AK Transition State and for the Nitrate Complex Ground State.

Entry	H-Bond Donor/Acceptor	AK TRANSITION STATE	N-(H)···O r_{DA}/ Å TS	(N)H·····O r_{DA}/ Å TS	N–H·····O angle /° TS	NITRATE GROUND STATE	N-(H)···O r_{DA}/ Å NO_3	(N)H····O r_{DA}/ Å NO_3	N–H····O angle /° NO_3
1	C271–R403	NH·······S	3.31	2.12	160.30	NH·······S	3.08	2.08	156.80
2	C271–T237	NH·······S	2.97	2.02	155.36	NH·······S	2.97	1.97	161.80
3	E224–Wat	C–O······Wat.Mg	2.83	1.93	154.98	C–O·····Wat.Mg	2.84	1.98	147.90
4	E224–Wat	C–O······Wat.Mg	2.72	1.8	159.72	C–O·····Wat.Mg	2.72	1.78	165.00
5	E225–Wat	Amide····Wat.Mg	2.73	1.79	165.9	Amide to Wat.Mg	2.8	1.9	171.2
6	E314(NH)–O2A	Amide····O–PA	2.95	2.01	151.86	Amide·····O–PA	3.06	2.14	150.50
7	E314O–Wat	C–O······Wat.Mg	2.82	1.87	169.17	C–O·····Wat.Mg	2.71	1.77	166.20
8	N274–E225	Amide·····O	3.21	2.35	141.56	Amide·····O	3.21	2.51	125.30
9	N274–O3G	Amide·····O	2.94	2.03	147.18	Amide·····O	3.21	2.42	134.30
10	Wat–O2G	Wat1220····O–PG	2.71	1.85	159.72	Wat1220····O–PG	2.89	2.09	138.10
11	R330–W1220	Bifurcated··Wat1220	3.14	2.38	130.18	Monodent··Wat1220	2.61	1.53	174.0
12	R330–W1220	Bifurcated··Wat1220	2.53	1.45	166.78	R330-N274	2.73	1.79	150.88
13	T273–W1220	ROH····Water	2.58	1.6	162.25	ROH····Water	2.55	1.70	137.80
14	R126–O2B	Bidentate	2.81	1.82	159.32	Bidentate	3.0	2.07	150.1
15	R126–O3B	Bidentate	2.85	1.84	166.03	Bidentate	2.77	1.80	159.50
16	R280–O3A	Bidentate	2.78	1.74	176.66	Bidentate	2.9	1.9	174.5
17	R280–O3B	Bidentate	2.9	1.88	165.9	Bidentate	2.58	1.51	170.30
18	R309–O1G	Bidentate	3.21	2.34	142.06	Bifurcated–O2G	3.33	2.44	144.80
19	R309–O2A	Bidentate	2.69	1.69	162.07	Bidentate	2.70	1.70	159.70
20	R309–O2G	Bidentate	2.73	1.81	146.55	Bifurcated–O2G	2.90	2.03	140.70
21	R403–E225	Bidentate	2.84	1.81	177.35	Bidentate	2.94	1.94	165.50
22	R403–E225	Bidentate	2.88	1.85	176.72	Bidentate	2.81	1.80	167.60
23	R403N2–E314O	Bidentate	2.85	1.81	176.0	Bidentate	2.80	1.80	164.10
24	R403Ne–E314O	Bidentate	2.79	1.76	176.33	Bidentate	2.98	1.96	170.20
25	R124–O2B	Bifurcated	2.93	2.09	138.27	Bidentate–O2B	2.59	1.52	170.90
26	R124–O2B	Bifurcated	2.69	1.69	157.72	*Absent*	n.a.	n.a.	n.a.
27	R126–N274	Bifurcated	2.76	1.81	152.28	Bifurcated	2.96	2.09	142.00
28	R126–N274	Bifurcated	2.86	1.95	146.01	Bifurcated	2.79	1.83	155.50
29	R229–O3G	Bifurcated	2.83	1.89	149.61	Bifurcated–O2B	2.89	1.98	146.40
30	R229–O3G	Bifurcated	2.81	1.91	144.46	Bifurcated	2.89	2.05	138.10

Supplementary Table S4. Summary of enlarged cluster model energetics.

Potential Energies in kcal/mol computed at different solvent dielectrics using the CPCM model

ε	React	TS	NPC	Prod
0	-5132483.5	-5132460.8	-5132497.4	-5132500.9
4	-5132638.8	-5132616.3	-5132636	-5132639.9
78	-5132696.6	-5132674.5	-5132687.3	-5132691.5

Potential Energy (U), Enthalpy (H) and Free Energy (G) in kcal/mol computed for the large Reactant, TS, NPC and Product cluster models. U+D corresponds to potential energy with Grimme's D3 dispersion correction. The energies shown were computed with CPCM solvation and ε=4.

Energy Type	React	TS	NPC	Prod
U	-5132638.8	-5132616.31	-5132636.0	-5132639.9
H	-5131093.3	-5131071.04	-5131093.7	-
G	-5131359.8	-5131334.41	-5131351.8	-
U+D	-5132674.3	-5132652.31	-5132671.7	-

Supplementary Table S5. Computed Bond Lengths and Angles for Arginine Kinase (Å and degrees).

Entry	Bond/Angle	Data from PDB 1m15	Mg·ADP·NO_3·Arg Complex	Mg·ADP·PO_3·Arg Small TS Complex	Mg·ADP·PO_3·Arg Large TS Complex
1	C5'-O5'	1.46	1.430	1.440	1.424
2	O5'-PA	1.56	1.617	1.616	1.600
3	PA-O1A	1.48	1.522	1.510	1.496
4	PA-O2A	1.51	1.521	1.522	1.497
5	PA-O3A	1.59	1.664	1.685	1.628
6	O3A-PB	1.65	1.669	1.65	1.643
7	PB-O1B	1.53	1.540	1.539	1.511
8	PB-O2B	1.48	1.542	1.543	1.528
9	PB-O3B	1.50	1.542	1.577	1.540
10	N'(PG)-O3B	3.23	2.790	2.076	2.198
11	N'(PG)-O1G	1.31	1.244	1.530	1.507
12	N'(PG)-O2G	1.37	1.243	1.526	1.494
13	N'(PG)-O3G	1.34	1.243	1.525	1.496
14	N'(PG)-N2	2.77	2.772	2.357	4.217
15	Mg-O1A	2.08	2.076	2.082	2.070
16	Mg-O1B	2.01	2.042	2.107	2.066
17	Mg-O1G(N')	2.10	2.212	2.056	2.068
18	O3B-N2	5.98	5.525	4.417	4.603
19	O3B-N'(PG)-N2˚	173.7˚	167.7	170.4˚	172.03˚
20	CD-N2-N'(PG)˚	112.3˚	119.7˚	116.6˚	113.3
21	CD-N2-H-H dihedral	n.a.	23.4˚	51.5˚	48.8˚
Hydrogen Bond Length to Substrate Arginine^{403} and (o.o.p. dihedral angle to guanidinium)					
22	C^{271}S–R^{403}1HH1		2.081 (19.4˚)	2.122 (1.9˚)	2.280 (7.7˚)
23	E^{225}OE1–R^{403}2HH1		1.804 (7.2˚)	1.812 (2.4˚)	1.811 (6.49˚)
24	E^{225}OE2–R^{403}2HH2		1.938 (18.0˚)	1.855 (19.7˚)	1.816 ()
25	E^{314}OE1–R^{403}HE		1.961 (2.9˚)	1.756 (2.1˚)	1.799 (7.9˚)
26	E^{314}OE2–R^{403}1HH2		1.800 (27.5˚)	1.813 (31.9˚)	1.817 (37.1˚)

H-bond lengths (N)H–O in Å.
o.o.p. dihedral angles O-N-CD-N averaged of the two angles
N_{nt} designates nitrogen from the NO_3^- group. n.a. = not available.

Supplementary Table S6. AK Final TS Model Data relative to 1m15 and computed results.

AK structure	N2–P_G / Å	P_G-O3B / Å	$\boldsymbol{r}_{DA}$ / Å (N2-O3B)	N2–P_G–O3B in-line angle
AK 1m15 nitrate complex	2.77	3.23	5.98	173.7˚
AK computed nitrate complex	2.77	2.79	5.26	167.7˚
AK initial TS	2.36	2.08	4.42	170.4˚
AK Final TS	2.42	2.20	4.60	172.0˚

Supplementary References

1. Yousef, M. S.; Fabiola, F.; Gattis, J. L.; Somasundaram, T.; Chapman, M. S. Refinement of the arginine kinase transition-state analogue complex at 1.2 Å resolution: mechanistic insights. *Acta Crystallogr. Sect. D; Struct. Biol.* **2002**, *58*, 2009-2017.

2. Himo, F. Recent trends in quantum chemical modeling of enzymatic reactions. *J. Am. Chem. Soc.* **2017**, *139*, 6780-6786.

3. Siegbahn, P. E. M.; Himo, F. The quantum chemical cluster approach for modeling enzyme reactions. *WIREs Comput. Mol. Sci.* **2011**, *1*, 323-336.

4. Jin, Y.; Molt, R. W., Jr.; Pellegrini, E.; Cliff, M. J.; Bowler, M. W.; Richards, N. G. J.; Blackburn, G. M. Assessing the influence of mutation on GTPase transition states using ^{19}F NMR, X-ray and DFT approaches. *Angew. Chem. Internat. Edn.* **2017**, *56*, 9732-9735.

5. Jin, Y.; Molt, R. W., Jr.; Waltho, J. P.; Richards, N. G. J.; Blackburn, G. M. ^{19}F NMR and DFT analysis reveal structural and electronic transition state features for RhoA-catalyzed GTP hydrolysis. *Angew. Chem. Internat. Edn.* **2016**, *55*, 3318-3322.

6. Zhao, Y.; Truhlar, D. G. The M06 suite of density functionals for main group thermochemistry, thermochemical kinetics, noncovalent interactions, excited states, and transition elements: two new functionals and systematic testing of four M06-class functionals and12 other functionals. *Theor. Chem. Acc.* **2008**, *120*, 215-241.

7. Mardirossian, N.; Head-Gordon, M. How accurate are the Minnesota density functionals for noncovalent interactions, isomerization energies, thermochemistry, and barrier heights involving molecules composed of main-group elements? *J. Chem. Theory Comput.* **2016**, *12*, 4303-4325.

8. Dunning, T. H., Jr. Gaussian-basis sets for use in correlated molecular calculations. 1. The atoms boron through neon and hydrogen. *J. Chem. Phys.* **1989**, *90*, 1007-1023.

9. Kendall, R. A; Dunning, T. H., Jr.; Harrison, R. J. Electron affinities of the first-row atoms revisited. Systematic basis sets and wave functions. *J. Chem. Phys.* **1992**, *96*, 6796-6806.

10. Atom names used in this paper are consistent with IUPAC nomenclature. See: Blackburn, G. M.; Cherfils, J.; Moss, G. P.; Richards, N. G. J.; Waltho, J. P.; Williams, N. H.; Wittinghofer, F. How to name atoms in phosphates, polyphosphates, their derivatives and mimics, and transition state analogues for enzyme-catalyzed phosphoryl transfer reactions (IUPAC Recommendations 2016). *Pure Appl. Chem.* **2017**, *89*, 653-675.

11. Johnson, B. G.; Frisch, M. J. Analytic 2nd derivatives of the gradient-corrected density-functional energy. Effect of quadrature weight derivatives. *Chem. Phys. Lett.* **1993**, *216*, 133-140.

12. Johnson, B. G.; Frisch, M. J. An implementation of analytic 2nd derivatives of the gradient-corrected density-functional energy. *J. Chem. Phys.* **1994**, *100*, 7429-7442.

13. Stratmann, R. E.; Burant, J. C.; Scuseria, G. E.; Frisch, M. J. Improving harmonic vibrational frequencies in density functional theory. *J. Chem. Phys.* **1997**, *106*, 10175-10183.

14. Gaussian 09, Revision D.01. Frisch, M. J.; Trucks, G. W.; Schlegel, H. B.; Scuseria, G. E.; Robb, M. A.; Cheeseman, J. R.; Scalmani, G.; Barone, V.; Mennucci, B.; Petersson, G. A.; Nakatsuji, H.; Caricato, M.; Li, X.; Hratchian, H. P.; Izmaylov, A. F.; Bloino, J.; Zheng, G.; Sonnenberg, J. L.; Hada, M.; Ehara, M.; Toyota, K.; Fukuda, R.; Hasegawa, J.; Ishida, M.; Nakajima, T.; Honda, Y.; Kitao, O.; Nakai, H.; Vreven, T.; Montgomery, J. A., Jr.; Peralta, J. E.; Ogliaro, F.; Bearpark, M.; Heyd, J. J.; Brothers, E.; Kudin, K. N.; Staroverov, V. N.; Keith, T.; Kobayashi, R.; Normand, J.; Raghavachari, K.; Rendell, A.; Burant, J. C.; Iyengar, S. S.; Tomasi, J.; Cossi, M.; Rega, N.; Millam, J. M.; Klene, M.; Knox, J. E.; Cross, J. B.; Bakken, V.; Adamo, C.; Jaramillo, J.; Gomperts, R.; Stratmann, R. E.; Yazyev, O.; Austin, A. J.; Cammi, R.; Pomelli, C.; Ochterski, J. W.; Martin, R. L.; Morokuma, K.; Zakrzewski, V. G.; Voth, G. A.; Salvador, P.; Dannenberg, J. J.; Dapprich, S.; Daniels, A. D.; Farkas, O.; Foresman, J. B.; Ortiz, J. V.; Cioslowski, J.; Fox, D. J. Gaussian, Inc., Wallingford CT, 2013.

15. Dennington, R.; Keith, T. A.; Millam, J. M. Semichem Inc., Shawnee Mission, KS, 2009.

16. Schlegel, H. B. An efficient algorithm for calculating *ab initio* energy gradients using S, P Cartesian Gaussians. *J. Chem. Phys.* **1982**, *77*, 3676-3681.

17. Gonzalez, C.; Schlegel, H. B. Reaction path following in mass-weighted internal coordinates. *J. Chem. Phys.* **1989**, *90,* 2154-2161.

18. Gaussian16, Revision B.01. Frisch, M. J.; Trucks, G. W.; Schlegel, H. B.; Scuseria, G. E.; Robb, M. A.; Cheeseman, J. R.; Scalmani, G.; Barone, V.; Petersson, G. A.; Nakatsuji, H.; Li, X.; Caricato, M.; Marenich, A. V.; Bloino, J.; Janesko, B. G.; Gomperts, R.; Mennucci, B.; Hratchian, H. P.; Ortiz, J. V.; Izmaylov, A. F.; Sonnenberg, J. L.; Williams-Young, D.; Ding, F.; Lipparini, F.; Egidi, F.; Goings, J.; Peng, B.; Petrone, A.; Henderson, T.; Ranasinghe, D.; Zakrzewski, V. G.; Gao, J.; Rega, N.; Zheng, G.; Liang, W.; Hada, M.; Ehara, M.; Toyota,

K.; Fukuda, R.; Hasegawa, J.; Ishida, M.; Nakajima, T.; Honda, Y.; Kitao, O.; Nakai, H.; Vreven, T.; Throssell, K.; Montgomery, J. A., Jr.; Peralta, J. E.; Ogliaro, F.; Bearpark, M. J.; Heyd, J. J.; Brothers, E. N.; Kudin, K. N.; Staroverov, V. N.; Keith, T. A.; Kobayashi, R.; Normand, J.; Raghavachari, K.; Rendell, A. P.; Burant, J. C.; Iyengar, S. S.; Tomasi, J.; Cossi, M.; Millam, J. M.; Klene, M.; Adamo, C.; Cammi, R.; Ochterski, J. W.; Martin, R. L.; Morokuma, K.; Farkas, O.; Foresman, J. B.; Fox, D. J. Gaussian, Inc., Wallingford CT, 2016.

19. Cossi, M.; Rega, N.; Scalmani, G.; Barone, V. Energies, structures, and electronic properties of molecules in solution with the C-PCM solvation model. *J. Comput. Chem.* **2003**, *24,* 669-681.

20. Schlegel, H. B. Optimization of equilibrium geometries and transition structures. *J. Comput. Chem.* **1982**, *3*, 214-218.

21. Grimme, S.; Anthony, J.; Ehrlich, S.; Krieg, H. A consistent and accurate *ab initio* parametrization of density functional dispersion correction (DFT-D) for the 94 elements HH-Pu. *J. Chem. Phys.* **2010**, *132*, 154104.

22. Davulcu, O.; Skalicky, J.J.; Chapman, M.S. Rate-limiting domaine and loop motions in arginine kinase *Biochemistry* **2011**, *50*, 4011-4018.

23. Travers, F.; Bertrand, R.; Roseau, G.; Van Thoai, N. Cryoenzymologic studies on arginine kinase: Solvent, temperature, and pH effects on the overall reaction. *Eur. J. Biochem.* **1978**. *88*, 523–528.

24. Suzuki, T.; Kanou, Y. 2014. Two distinct arginine kinases in *Neocaridina denticulate*: Psychrophilic and mesophilic enzymes. *Int. J. Biol. Macromol.* **2014**, *67*, 433-438.

25. Suzuki, T.; Yamamoto, K.; Tada, H.; Uda, K., Cold-adapted features of arginine kinase from the deep-sea clam *Calyptogena kaikoi*. *Marine Biotech.* **2012**, *14*, 294-303.

26. Briggs, R.W.; Radda, G.K.; Thulborn, K.R. ^{31}P-NMR saturation transfer study of the *in vivo* kinetics of arginine kinase in *Carcinus* crab leg muscle. *Biochim. Biophys. Acta Mol. Cell Res.* **1985**, *845*, 343-348.

6. Coordinate Data for the Cluster Models

Small AK-nitrate Cluster Model (229 atoms, charge +1)

ATOM	1	CG	ARG	A	124	8.324	5.443	-1.077	C
ATOM	2	CD	ARG	A	124	8.395	3.947	-0.761	C
ATOM	3	NE	ARG	A	124	7.605	3.098	-1.650	N
ATOM	4	CZ	ARG	A	124	6.292	2.848	-1.491	C
ATOM	5	NH1	ARG	A	124	5.599	3.442	-0.526	N
ATOM	6	NH2	ARG	A	124	5.665	1.998	-2.307	N
ATOM	7	CG	ARG	A	126	3.054	4.760	-2.681	C
ATOM	8	CD	ARG	A	126	1.528	4.772	-2.693	C
ATOM	9	NE	ARG	A	126	1.000	4.018	-1.563	N
ATOM	10	CZ	ARG	A	126	-0.308	3.857	-1.350	C
ATOM	11	NH1	ARG	A	126	-0.759	3.126	-0.324	N
ATOM	12	NH2	ARG	A	126	-1.197	4.520	-2.111	N
ATOM	13	CG	GLU	A	224	1.549	-5.722	-6.136	C
ATOM	14	CD	GLU	A	224	1.566	-4.591	-5.076	C
ATOM	15	OE1	GLU	A	224	0.741	-3.652	-5.137	O
ATOM	16	OE2	GLU	A	224	2.453	-4.661	-4.159	O
ATOM	17	CG	GLU	A	225	-3.676	-3.174	-4.437	C
ATOM	18	CD	GLU	A	225	-3.326	-2.304	-3.255	C
ATOM	19	OE1	GLU	A	225	-4.125	-1.387	-2.906	O
ATOM	20	OE2	GLU	A	225	-2.226	-2.514	-2.670	O
ATOM	21	CG	ARG	A	229	1.225	0.879	-6.799	C
ATOM	22	CD	ARG	A	229	0.316	1.927	-6.171	C
ATOM	23	NE	ARG	A	229	-0.848	1.362	-5.497	N
ATOM	24	CZ	ARG	A	229	-0.869	1.032	-4.190	C
ATOM	25	NH1	ARG	A	229	0.165	1.317	-3.386	N
ATOM	26	NH2	ARG	A	229	-1.977	0.491	-3.701	N
ATOM	27	CB	THR	A	273	-7.092	2.380	1.984	C
ATOM	28	OG1	THR	A	273	-6.262	2.344	0.825	O
ATOM	29	CB	ASN	A	274	-5.345	3.192	-2.761	C
ATOM	30	CG	ASN	A	274	-4.341	2.648	-1.786	C
ATOM	31	OD1	ASN	A	274	-3.577	3.425	-1.150	O
ATOM	32	ND2	ASN	A	274	-4.289	1.339	-1.612	N
ATOM	33	CG	ARG	A	280	1.320	5.524	2.186	C
ATOM	34	CD	ARG	A	280	0.824	4.204	2.773	C
ATOM	35	NE	ARG	A	280	1.820	3.148	2.681	N
ATOM	36	CZ	ARG	A	280	2.867	3.056	3.469	C
ATOM	37	NH1	ARG	A	280	2.980	3.802	4.600	N
ATOM	38	NH2	ARG	A	280	3.870	2.212	3.176	N
ATOM	39	CD	ARG	A	309	2.124	0.633	5.774	C
ATOM	40	NE	ARG	A	309	0.758	0.944	5.366	N
ATOM	41	CZ	ARG	A	309	0.177	0.548	4.213	C
ATOM	42	NH1	ARG	A	309	0.860	0.052	3.217	N
ATOM	43	NH2	ARG	A	309	-1.167	0.779	4.066	N
ATOM	44	CA	GLY	A	313	5.514	-2.739	4.613	C
ATOM	45	C	GLY	A	313	4.436	-3.133	5.599	C
ATOM	46	O	GLY	A	313	4.638	-3.841	6.567	O
ATOM	47	N	GLU	A	314	3.189	-2.648	5.304	N
ATOM	48	CA	GLU	A	314	2.030	-3.245	5.952	C
ATOM	49	CB	GLU	A	314	0.747	-2.859	5.221	C
ATOM	50	CG	GLU	A	314	0.719	-3.304	3.763	C
ATOM	51	CD	GLU	A	314	-0.550	-2.938	3.002	C
ATOM	52	OE1	GLU	A	314	-1.315	-2.059	3.459	O
ATOM	53	OE2	GLU	A	314	-0.734	-3.554	1.903	O
ATOM	54	CG	ARG	A	330	-4.552	7.574	0.922	C
ATOM	55	CD	ARG	A	330	-3.547	6.563	0.356	C
ATOM	56	NE	ARG	A	330	-3.601	5.218	0.941	N
ATOM	57	CZ	ARG	A	330	-2.901	4.845	2.009	C

```
ATOM     58  NH1 ARG A 330      -2.260   5.807   2.746                       N
ATOM     59  NH2 ARG A 330      -2.774   3.574   2.347                       N
HETATM   60  O1  NO3 A 405       0.112  -1.011   0.359                       O
HETATM   61  O2  NO3 A 405      -1.299   0.398   1.152                       O
HETATM   62  O3  NO3 A 405      -1.067   0.212  -0.978                       O
HETATM   63 MG    MG A 408       1.542  -1.778  -1.053                      Mg
ATOM     64  CA  ARG A 403      -6.070  -5.372   3.635                       C
ATOM     65  CB  ARG A 403      -5.176  -4.135   3.498                       C
ATOM     66  CG  ARG A 403      -5.858  -3.007   2.725                       C
ATOM     67  CD  ARG A 403      -5.108  -1.674   2.741                       C
ATOM     68  NE  ARG A 403      -3.803  -1.684   2.069                       N
ATOM     69  CZ  ARG A 403      -3.690  -1.839   0.729                       C
ATOM     70  NH1 ARG A 403      -4.674  -1.467  -0.083                       N
ATOM     71  NH2 ARG A 403      -2.553  -2.316   0.230                       N
HETATM   72  PB  ADP A 400       2.511   1.192  -0.216                       P
HETATM   73  O1B ADP A 400       2.164   0.168  -1.322                       O
HETATM   74  O2B ADP A 400       3.184   2.465  -0.783                       O
HETATM   75  O3B ADP A 400       1.334   1.559   0.725                       O
HETATM   76  PA  ADP A 400       3.689  -0.976   1.431                       P
HETATM   77  O1A ADP A 400       2.828  -1.876   0.568                       O
HETATM   78  O2A ADP A 400       3.410  -0.786   2.918                       O
HETATM   79  O3A ADP A 400       3.690   0.559   0.764                       O
HETATM   80  O5P ADP A 400       5.260  -1.302   1.278                       O
HETATM   81  C5P ADP A 400       5.703  -1.452  -0.077                       C
ATOM     82  CB  CYS A 271      -7.569  -0.965  -1.589                       C
ATOM     83  SG  CYS A 271      -7.542  -0.189   0.060                       S
HETATM   84  O   HOH A1003       3.277  -6.350  -2.388                       O
HETATM   85  O   HOH A1210       3.008   1.348  -3.652                       O
HETATM   86  O   HOH A1212      -4.709   0.461  -4.625                       O
HETATM   87  O   HOH A1214       4.766  -0.766  -3.299                       O
HETATM   88  O   HOH A1215       4.281  -4.410  -0.734                       O
HETATM   89  O   HOH A1216      -0.551  -6.151   0.760                       O
HETATM   90  O   HOH A1219      -1.185  -5.002  -1.721                       O
HETATM   91  O   HOH A1220      -3.875   1.496   1.235                       O
HETATM   92  O   HOH A1223       1.981  -5.776  -0.004                       O
HETATM   93  O   HOH A1282      -1.035  -1.558  -5.685                       O
HETATM   94  O   HOH A1296       2.982  -2.710  -2.240                       O
HETATM   95  O   HOH A1297       0.765  -3.519  -0.363                       O
HETATM   96  O   HOH A1298       0.387  -1.865  -2.799                       O
ATOM     97 1HG  ARG A 124       7.295   5.825  -1.013                       H
ATOM     98 2HG  ARG A 124       8.693   5.644  -2.093                       H
ATOM     99 1HD  ARG A 124       8.069   3.740   0.269                       H
ATOM    100 2HD  ARG A 124       9.433   3.595  -0.827                       H
ATOM    101  HE  ARG A 124       8.035   2.751  -2.499                       H
ATOM    102 1HH1 ARG A 124       5.986   4.241  -0.046                       H
ATOM    103 2HH1 ARG A 124       4.596   3.138  -0.419                       H
ATOM    104 1HH2 ARG A 124       4.648   1.893  -2.226                       H
ATOM    105 2HH2 ARG A 124       6.151   1.251  -2.790                       H
ATOM    106 1HG  ARG A 126       3.434   5.097  -1.707                       H
ATOM    107 2HG  ARG A 126       3.425   3.748  -2.885                       H
ATOM    108 1HD  ARG A 126       1.151   5.807  -2.638                       H
ATOM    109 2HD  ARG A 126       1.165   4.329  -3.638                       H
ATOM    110  HE  ARG A 126       1.681   3.408  -1.084                       H
ATOM    111 1HH1 ARG A 126      -0.102   2.450   0.095                       H
ATOM    112 2HH1 ARG A 126      -1.743   2.842  -0.369                       H
ATOM    113 1HH2 ARG A 126      -2.181   4.251  -1.987                       H
ATOM    114 2HH2 ARG A 126      -0.896   4.868  -3.011                       H
ATOM    115 1HG  GLU A 224       2.558  -5.840  -6.551                       H
ATOM    116 2HG  GLU A 224       1.272  -6.661  -5.636                       H
ATOM    117 1HG  GLU A 225      -3.111  -2.789  -5.301                       H
ATOM    118 2HG  GLU A 225      -4.750  -3.145  -4.648                       H
ATOM    119 1HG  ARG A 229       1.637   0.210  -6.029                       H
ATOM    120 2HG  ARG A 229       0.668   0.260  -7.516                       H
```

```
ATOM    121 1HD  ARG A 229       0.869   2.539  -5.444                          H
ATOM    122 2HD  ARG A 229      -0.061   2.616  -6.941                          H
ATOM    123  HE  ARG A 229      -1.403   0.716  -6.059                          H
ATOM    124 1HH2 ARG A 229      -2.003   0.123  -2.751                          H
ATOM    125 2HH2 ARG A 229      -2.849   0.495  -4.240                          H
ATOM    126 1HH1 ARG A 229       0.122   0.888  -2.456                          H
ATOM    127 2HH1 ARG A 229       1.105   1.317  -3.795                          H
ATOM    128  HB  THR A 273      -7.289   1.372   2.383                          H
ATOM    129  HG1 THR A 273      -6.661   1.604   0.261                          H
ATOM    130 1HB  ASN A 274      -5.526   2.476  -3.573                          H
ATOM    131 2HB  ASN A 274      -4.994   4.152  -3.156                          H
ATOM    132 1HD2 ASN A 274      -3.716   0.969  -0.851                          H
ATOM    133 2HD2 ASN A 274      -4.889   0.704  -2.131                          H
ATOM    134 1HG  ARG A 280       0.530   6.286   2.243                          H
ATOM    135 2HG  ARG A 280       2.209   5.897   2.718                          H
ATOM    136 1HD  ARG A 280       0.491   4.318   3.821                          H
ATOM    137 2HD  ARG A 280      -0.045   3.846   2.202                          H
ATOM    138  HE  ARG A 280       1.688   2.434   1.864                          H
ATOM    139 1HH2 ARG A 280       4.429   1.843   3.937                          H
ATOM    140 2HH2 ARG A 280       3.747   1.564   2.391                          H
ATOM    141 1HH1 ARG A 280       3.908   3.939   4.981                          H
ATOM    142 2HH1 ARG A 280       2.334   4.568   4.738                          H
ATOM    143 1HD  ARG A 309       2.507   1.463   6.381                          H
ATOM    144 2HD  ARG A 309       2.757   0.515   4.885                          H
ATOM    145  HE  ARG A 309       0.113   1.198   6.105                          H
ATOM    146 1HH1 ARG A 309       1.870  -0.188   3.236                          H
ATOM    147 2HH1 ARG A 309       0.347  -0.211   2.368                          H
ATOM    148 1HH2 ARG A 309      -1.691   0.562   4.911                          H
ATOM    149 2HH2 ARG A 309      -1.535   0.213   3.290                          H
ATOM    150 1HA  GLY A 313       5.399  -1.700   4.275                          H
ATOM    151 2HA  GLY A 313       5.435  -3.379   3.722                          H
ATOM    152 1HB  GLU A 314      -0.109  -3.305   5.750                          H
ATOM    153 2HB  GLU A 314       0.595  -1.769   5.276                          H
ATOM    154 1HG  GLU A 314       0.860  -4.392   3.682                          H
ATOM    155 2HG  GLU A 314       1.545  -2.857   3.178                          H
ATOM    156  HA  GLU A 314       1.987  -2.921   7.004                          H
ATOM    157  H   GLU A 314       3.078  -2.222   4.386                          H
ATOM    158 1HG  ARG A 330      -4.392   7.775   1.992                          H
ATOM    159 2HG  ARG A 330      -5.579   7.203   0.805                          H
ATOM    160 1HD  ARG A 330      -2.517   6.946   0.439                          H
ATOM    161 2HD  ARG A 330      -3.737   6.416  -0.715                          H
ATOM    162  HE  ARG A 330      -3.895   4.458   0.310                          H
ATOM    163 1HH1 ARG A 330      -2.045   5.548   3.702                          H
ATOM    164 2HH1 ARG A 330      -2.635   6.746   2.667                          H
ATOM    165 1HH2 ARG A 330      -2.231   3.335   3.169                          H
ATOM    166 2HH2 ARG A 330      -3.218   2.736   1.827                          H
ATOM    167 1HB  ARG A 403      -4.906  -3.768   4.504                          H
ATOM    168 2HB  ARG A 403      -4.227  -4.412   3.006                          H
ATOM    169 1HG  ARG A 403      -6.850  -2.803   3.157                          H
ATOM    170 2HG  ARG A 403      -6.029  -3.311   1.677                          H
ATOM    171 1HD  ARG A 403      -4.919  -1.365   3.779                          H
ATOM    172 2HD  ARG A 403      -5.742  -0.892   2.294                          H
ATOM    173  HE  ARG A 403      -2.977  -1.995   2.603                          H
ATOM    174 1HH1 ARG A 403      -5.579  -1.058   0.237                          H
ATOM    175 2HH1 ARG A 403      -4.515  -1.548  -1.091                          H
ATOM    176 1HH2 ARG A 403      -1.865  -2.759   0.860                          H
ATOM    177 2HH2 ARG A 403      -2.429  -2.362  -0.782                          H
ATOM    178  HA  ARG A 403      -5.563  -6.175   4.189                          H
ATOM    179 1HB  CYS A 271      -6.557  -1.204  -1.955                          H
ATOM    180 2HB  CYS A 271      -8.139  -1.903  -1.554                          H
HETATM  181 1H5P ADP A 400       5.358  -0.614  -0.700                          H
HETATM  182 2H5P ADP A 400       5.344  -2.406  -0.486                          H
HETATM  183 1H   HOH A1003       3.616  -7.171  -2.765                          H
```

```
HETATM  184 2H   HOH A1003       2.938  -5.813  -3.163                       H
HETATM  185 1H   HOH A1210       2.727   0.965  -2.791                       H
HETATM  186 2H   HOH A1210       3.605   0.643  -3.963                       H
HETATM  187 1H   HOH A1212      -4.690  -0.355  -4.060                       H
HETATM  188 2H   HOH A1212      -5.195   0.203  -5.419                       H
HETATM  189 1H   HOH A1214       5.178  -1.398  -3.905                       H
HETATM  190 2H   HOH A1214       4.113  -1.333  -2.826                       H
HETATM  191 1H   HOH A1215       4.169  -5.205  -1.301                       H
HETATM  192 2H   HOH A1215       3.678  -4.607   0.006                       H
HETATM  193 1H   HOH A1216      -1.043  -5.917  -0.051                       H
HETATM  194 2H   HOH A1216      -0.714  -5.393   1.352                       H
HETATM  195 1H   HOH A1219      -0.491  -4.394  -1.408                       H
HETATM  196 2H   HOH A1219      -1.754  -4.414  -2.240                       H
HETATM  197 1H   HOH A1220      -3.738   0.593   1.569                       H
HETATM  198 2H   HOH A1220      -4.862   1.682   1.117                       H
HETATM  199 1H   HOH A1223       2.188  -6.225  -0.847                       H
HETATM  200 2H   HOH A1223       1.110  -6.128   0.320                       H
HETATM  201 1H   HOH A1282      -0.479  -2.335  -5.885                       H
HETATM  202 2H   HOH A1282      -1.092  -1.617  -4.719                       H
HETATM  203  H   HOH A1296       3.509  -3.319  -1.631                       H
HETATM  204  H   HOH A1297       0.323  -3.437   0.518                       H
HETATM  205  H   HOH A1298      -0.571  -2.035  -2.642                       H
HETATM  206  O             0    -8.061  -0.759   3.121                       O
HETATM  207  H             0    -8.978  -0.485   3.254                       H
HETATM  208  H             0    -8.018  -0.894   2.151                       H
HETATM  209  H             0     1.324  -4.352  -0.279                       H
HETATM  210  H             0     0.629  -2.514  -3.504                       H
HETATM  211  H             0     2.691  -3.318  -2.978                       H
HETATM  212  H             0     8.947   6.003  -0.367                       H
HETATM  213  H             0     6.494  -2.897   5.075                       H
HETATM  214  H             0     2.150  -4.341   5.975                       H
HETATM  215  H             0    -7.000  -5.121   4.167                       H
HETATM  216  H             0    -6.347  -5.766   2.646                       H
HETATM  217  H             0     3.423   5.442  -3.458                       H
HETATM  218  H             0     6.799  -1.455  -0.042                       H
HETATM  219  H             0     2.056   1.368  -7.326                       H
HETATM  220  H             0    -4.456   8.525   0.381                       H
HETATM  221  H             0    -6.576   2.978   2.751                       H
HETATM  222  H             0    -8.055   2.863   1.754                       H
HETATM  223  H             0    -8.050  -0.301  -2.321                       H
HETATM  224  H             0    -6.284   3.357  -2.210                       H
HETATM  225  H             0     0.826  -5.499  -6.927                       H
HETATM  226  H             0     1.587   5.376   1.130                       H
HETATM  227  H             0    -3.341  -4.204  -4.263                       H
HETATM  228  H             0     2.181  -0.308   6.341                       H
HETATM  229  N             0    -0.760  -0.131   0.175                       N
```

Small TS Cluster Model (229 atoms, charge +1)

```
ATOM      1  CG  ARG A 124       7.992   5.903  -1.369                       C
ATOM      2  CD  ARG A 124       7.954   4.451  -0.884                       C
ATOM      3  NE  ARG A 124       7.171   3.541  -1.724                       N
ATOM      4  CZ  ARG A 124       5.899   3.172  -1.497                       C
ATOM      5  NH1 ARG A 124       5.172   3.782  -0.555                       N
ATOM      6  NH2 ARG A 124       5.336   2.190  -2.196                       N
ATOM      7  CG  ARG A 126       2.798   4.759  -2.960                       C
ATOM      8  CD  ARG A 126       1.277   4.740  -3.049                       C
ATOM      9  NE  ARG A 126       0.712   4.065  -1.889                       N
ATOM     10  CZ  ARG A 126      -0.597   3.994  -1.660                       C
ATOM     11  NH1 ARG A 126      -1.072   3.368  -0.569                       N
ATOM     12  NH2 ARG A 126      -1.462   4.640  -2.462                       N
ATOM     13  CG  GLU A 224       2.043  -6.005  -5.722                       C
```

ATOM	14	CD	GLU	A	224	1.991	-4.826	-4.733	C
ATOM	15	OE1	GLU	A	224	1.109	-3.944	-4.850	O
ATOM	16	OE2	GLU	A	224	2.875	-4.793	-3.815	O
ATOM	17	CG	GLU	A	225	-3.360	-3.713	-4.232	C
ATOM	18	CD	GLU	A	225	-3.094	-2.803	-3.054	C
ATOM	19	OE1	GLU	A	225	-3.917	-1.877	-2.791	O
ATOM	20	OE2	GLU	A	225	-2.038	-2.984	-2.387	O
ATOM	21	CG	ARG	A	229	1.282	0.501	-6.824	C
ATOM	22	CD	ARG	A	229	0.143	1.446	-6.471	C
ATOM	23	NE	ARG	A	229	-0.936	0.784	-5.751	N
ATOM	24	CZ	ARG	A	229	-0.942	0.626	-4.414	C
ATOM	25	NH1	ARG	A	229	-0.025	1.215	-3.629	N
ATOM	26	NH2	ARG	A	229	-1.921	-0.087	-3.874	N
ATOM	27	CB	THR	A	273	-7.216	2.005	1.784	C
ATOM	28	OG1	THR	A	273	-6.263	1.852	0.731	O
ATOM	29	CB	ASN	A	274	-5.475	2.621	-2.993	C
ATOM	30	CG	ASN	A	274	-4.323	2.420	-2.059	C
ATOM	31	OD1	ASN	A	274	-3.756	3.409	-1.521	O
ATOM	32	ND2	ASN	A	274	-3.944	1.172	-1.830	N
ATOM	33	CG	ARG	A	280	0.962	5.721	1.835	C
ATOM	34	CD	ARG	A	280	0.360	4.443	2.412	C
ATOM	35	NE	ARG	A	280	1.303	3.332	2.437	N
ATOM	36	CZ	ARG	A	280	2.368	3.299	3.227	C
ATOM	37	NH1	ARG	A	280	2.504	4.215	4.213	N
ATOM	38	NH2	ARG	A	280	3.339	2.415	3.026	N
ATOM	39	CD	ARG	A	309	2.054	1.142	5.744	C
ATOM	40	NE	ARG	A	309	0.712	1.434	5.274	N
ATOM	41	CZ	ARG	A	309	0.116	0.862	4.204	C
ATOM	42	NH1	ARG	A	309	0.797	0.271	3.255	N
ATOM	43	NH2	ARG	A	309	-1.232	1.022	4.090	N
ATOM	44	CA	GLY	A	313	5.677	-2.060	4.832	C
ATOM	45	C	GLY	A	313	4.617	-2.383	5.864	C
ATOM	46	O	GLY	A	313	4.865	-2.866	6.953	O
ATOM	47	N	GLU	A	314	3.335	-2.109	5.465	N
ATOM	48	CA	GLU	A	314	2.219	-2.713	6.177	C
ATOM	49	CB	GLU	A	314	0.921	-2.508	5.399	C
ATOM	50	CG	GLU	A	314	0.929	-3.120	3.999	C
ATOM	51	CD	GLU	A	314	-0.323	-2.848	3.168	C
ATOM	52	OE1	GLU	A	314	-1.114	-1.945	3.539	O
ATOM	53	OE2	GLU	A	314	-0.468	-3.538	2.112	O
ATOM	54	CG	ARG	A	330	-5.021	7.279	0.397	C
ATOM	55	CD	ARG	A	330	-3.926	6.310	-0.061	C
ATOM	56	NE	ARG	A	330	-3.811	5.097	0.747	N
ATOM	57	CZ	ARG	A	330	-3.216	5.043	1.936	C
ATOM	58	NH1	ARG	A	330	-2.700	6.191	2.477	N
ATOM	59	NH2	ARG	A	330	-3.085	3.892	2.567	N
HETATM	60	O1	PO3	A	405	0.388	-1.128	0.393	O
HETATM	61	O2	PO3	A	405	-1.436	0.512	1.404	O
HETATM	62	O3	PO3	A	405	-1.193	0.319	-1.176	O
HETATM	63	MG	MG	A	408	1.703	-1.896	-0.999	Mg
ATOM	64	CA	ARG	A	403	-5.691	-5.534	3.952	C
ATOM	65	CB	ARG	A	403	-4.843	-4.276	3.740	C
ATOM	66	CG	ARG	A	403	-5.563	-3.219	2.902	C
ATOM	67	CD	ARG	A	403	-4.849	-1.867	2.837	C
ATOM	68	NE	ARG	A	403	-3.552	-1.907	2.162	N
ATOM	69	CZ	ARG	A	403	-3.407	-1.877	0.844	C
ATOM	70	NH1	ARG	A	403	-4.402	-1.630	0.008	N
ATOM	71	NH2	ARG	A	403	-2.143	-2.050	0.333	N
HETATM	72	PB	ADP	A	400	2.188	1.193	-0.367	P
HETATM	73	O1B	ADP	A	400	2.179	0.102	-1.453	O
HETATM	74	O2B	ADP	A	400	2.762	2.558	-0.813	O
HETATM	75	O3B	ADP	A	400	0.800	1.436	0.335	O
HETATM	76	PA	ADP	A	400	3.656	-0.774	1.441	P

```
HETATM   77  O1A ADP A 400       3.110  -1.849   0.532                          O
HETATM   78  O2A ADP A 400       3.314  -0.637   2.918                          O
HETATM   79  O3A ADP A 400       3.251   0.738   0.817                          O
HETATM   80  O5P ADP A 400       5.266  -0.701   1.335                          O
HETATM   81  C5P ADP A 400       5.831  -1.074   0.068                          C
ATOM     82  CB  CYS A 271      -7.426  -1.592  -1.563                          C
ATOM     83  SG  CYS A 271      -7.428  -0.798   0.082                          S
HETATM   84  O   HOH A1003       3.832  -6.347  -1.956                          O
HETATM   85  O   HOH A1210       2.835   1.394  -3.754                          O
HETATM   86  O   HOH A1212      -4.720  -0.251  -4.610                          O
HETATM   87  O   HOH A1214       4.791  -0.535  -3.241                          O
HETATM   88  O   HOH A1215       4.685  -4.159  -0.521                          O
HETATM   89  O   HOH A1216       0.032  -6.178   1.235                          O
HETATM   90  O   HOH A1219      -0.867  -5.351  -1.301                          O
HETATM   91  O   HOH A1220      -3.707   1.939   1.094                          O
HETATM   92  O   HOH A1223       2.539  -5.711   0.421                          O
HETATM   93  O   HOH A1282      -0.774  -2.107  -5.705                          O
HETATM   94  O   HOH A1296       3.239  -2.672  -2.160                          O
HETATM   95  O   HOH A1297       1.084  -3.663  -0.212                          O
HETATM   96  O   HOH A1298       0.528  -2.161  -2.722                          O
ATOM     97 1HG  ARG A 124       6.985   6.345  -1.400                          H
ATOM     98 2HG  ARG A 124       8.421   5.968  -2.378                          H
ATOM     99 1HD  ARG A 124       7.552   4.380   0.137                          H
ATOM    100 2HD  ARG A 124       8.973   4.043  -0.838                          H
ATOM    101  HE  ARG A 124       7.624   3.122  -2.528                          H
ATOM    102 1HH1 ARG A 124       5.430   4.711  -0.255                          H
ATOM    103 2HH1 ARG A 124       4.201   3.429  -0.434                          H
ATOM    104 1HH2 ARG A 124       4.327   2.035  -2.101                          H
ATOM    105 2HH2 ARG A 124       5.855   1.460  -2.673                          H
ATOM    106 1HG  ARG A 126       3.119   5.199  -2.005                          H
ATOM    107 2HG  ARG A 126       3.189   3.737  -3.048                          H
ATOM    108 1HD  ARG A 126       0.883   5.770  -3.087                          H
ATOM    109 2HD  ARG A 126       0.967   4.220  -3.973                          H
ATOM    110  HE  ARG A 126       1.368   3.479  -1.340                          H
ATOM    111 1HH1 ARG A 126      -0.458   2.637  -0.189                          H
ATOM    112 2HH1 ARG A 126      -2.071   3.128  -0.613                          H
ATOM    113 1HH2 ARG A 126      -2.445   4.359  -2.354                          H
ATOM    114 2HH2 ARG A 126      -1.144   4.910  -3.384                          H
ATOM    115 1HB  GLU A 224       3.064  -6.106  -6.113                          H
ATOM    116 2HB  GLU A 224       1.795  -6.926  -5.176                          H
ATOM    117 1HG  GLU A 225      -2.788  -3.313  -5.085                          H
ATOM    118 2HG  GLU A 225      -4.428  -3.739  -4.479                          H
ATOM    119 1HG  ARG A 229       1.753   0.102  -5.913                          H
ATOM    120 2HG  ARG A 229       0.908  -0.352  -7.407                          H
ATOM    121 1HD  ARG A 229       0.497   2.287  -5.857                          H
ATOM    122 2HD  ARG A 229      -0.294   1.882  -7.380                          H
ATOM    123  HE  ARG A 229      -1.337  -0.026  -6.226                          H
ATOM    124 1HH2 ARG A 229      -1.899  -0.261  -2.866                          H
ATOM    125 2HH2 ARG A 229      -2.797  -0.227  -4.384                          H
ATOM    126 1HH1 ARG A 229      -0.100   0.935  -2.643                          H
ATOM    127 2HH1 ARG A 229       0.929   1.311  -3.990                          H
ATOM    128 1HB  THR A 273      -7.363   1.066   2.339                          H
ATOM    129  HG1 THR A 273      -6.568   1.022   0.239                          H
ATOM    130 1HB  ASN A 274      -5.551   1.794  -3.709                          H
ATOM    131 2HB  ASN A 274      -5.363   3.582  -3.508                          H
ATOM    132 1HD2 ASN A 274      -3.129   0.969  -1.248                          H
ATOM    133 2HD2 ASN A 274      -4.382   0.400  -2.328                          H
ATOM    134 1HG  ARG A 280       0.204   6.516   1.831                          H
ATOM    135 2HG  ARG A 280       1.831   6.065   2.413                          H
ATOM    136 1HD  ARG A 280      -0.042   4.610   3.430                          H
ATOM    137 2HD  ARG A 280      -0.485   4.107   1.799                          H
ATOM    138  HE  ARG A 280       1.151   2.551   1.769                          H
ATOM    139 1HH2 ARG A 280       3.989   2.184   3.767                          H
```

```
ATOM    140 2HH2 ARG A 280       3.292   1.765   2.226                           H
ATOM    141 1HH1 ARG A 280       3.318   4.183   4.812                           H
ATOM    142 2HH1 ARG A 280       1.689   4.703   4.557                           H
ATOM    143 1HD  ARG A 309       2.580   2.079   5.977                           H
ATOM    144 2HD  ARG A 309       2.617   0.587   4.982                           H
ATOM    145  HE  ARG A 309       0.068   1.833   5.946                           H
ATOM    146 1HH1 ARG A 309       1.813   0.053   3.267                           H
ATOM    147 2HH1 ARG A 309       0.245  -0.259   2.576                           H
ATOM    148 1HH2 ARG A 309      -1.742   0.770   4.933                           H
ATOM    149 2HH2 ARG A 309      -1.593   0.551   3.248                           H
ATOM    150 1HA  GLY A 313       5.434  -1.151   4.264                           H
ATOM    151 2HA  GLY A 313       5.730  -2.890   4.111                           H
ATOM    152 1HB  GLU A 314       0.089  -2.939   5.975                           H
ATOM    153 2HB  GLU A 314       0.705  -1.430   5.321                           H
ATOM    154 1HG  GLU A 314       1.077  -4.209   4.045                           H
ATOM    155 2HG  GLU A 314       1.764  -2.735   3.385                           H
ATOM    156 1HA  GLU A 314       2.142  -2.268   7.181                           H
ATOM    157 1HA  GLU A 314       3.216  -1.883   4.480                           H
ATOM    158 1HG  ARG A 330      -4.852   7.675   1.410                           H
ATOM    159 2HG  ARG A 330      -5.997   6.776   0.399                           H
ATOM    160 1HD  ARG A 330      -2.943   6.810  -0.107                           H
ATOM    161 2HD  ARG A 330      -4.144   5.951  -1.074                           H
ATOM    162  HE  ARG A 330      -4.025   4.205   0.282                           H
ATOM    163 1HH1 ARG A 330      -2.607   6.190   3.487                           H
ATOM    164 2HH1 ARG A 330      -3.066   7.064   2.114                           H
ATOM    165 1HH2 ARG A 330      -2.583   3.866   3.445                           H
ATOM    166 2HH2 ARG A 330      -3.362   2.967   2.032                           H
ATOM    167 1HB  ARG A 403      -4.589  -3.840   4.722                           H
ATOM    168 2HB  ARG A 403      -3.884  -4.546   3.265                           H
ATOM    169 1HG  ARG A 403      -6.559  -3.020   3.326                           H
ATOM    170 2HG  ARG A 403      -5.726  -3.589   1.874                           H
ATOM    171 1HD  ARG A 403      -4.662  -1.497   3.855                           H
ATOM    172 2HD  ARG A 403      -5.497  -1.126   2.343                           H
ATOM    173  HE  ARG A 403      -2.679  -2.026   2.709                           H
ATOM    174 1HH1 ARG A 403      -5.386  -1.413   0.297                           H
ATOM    175 2HH1 ARG A 403      -4.207  -1.687  -0.998                           H
ATOM    176 1HH2 ARG A 403      -1.531  -2.618   0.943                           H
ATOM    177 2HH2 ARG A 403      -2.129  -2.351  -0.651                           H
ATOM    178 1HA  ARG A 403      -5.156  -6.283   4.552                           H
ATOM    179 1HB  CYS A 271      -6.411  -1.901  -1.863                           H
ATOM    180 2HB  CYS A 271      -8.062  -2.487  -1.551                           H
HETATM  181 1H5P ADP A 400       5.395  -0.476  -0.747                           H
HETATM  182 2H5P ADP A 400       5.663  -2.141  -0.128                           H
HETATM  183 1H   HOH A1003       4.249  -7.146  -2.301                           H
HETATM  184 2H   HOH A1003       3.449  -5.877  -2.748                           H
HETATM  185 1H   HOH A1210       2.575   0.916  -2.933                           H
HETATM  186 2H   HOH A1210       3.530   0.796  -4.079                           H
HETATM  187 1H   HOH A1212      -4.642  -1.022  -3.988                           H
HETATM  188 2H   HOH A1212      -5.349  -0.530  -5.287                           H
HETATM  189 1H   HOH A1214       5.300  -1.165  -3.769                           H
HETATM  190 2H   HOH A1214       4.166  -1.133  -2.767                           H
HETATM  191 1H   HOH A1215       4.631  -5.019  -0.991                           H
HETATM  192 2H   HOH A1215       4.088  -4.302   0.235                           H
HETATM  193 1H   HOH A1216      -0.508  -6.090   0.425                           H
HETATM  194 2H   HOH A1216      -0.194  -5.371   1.736                           H
HETATM  195 1H   HOH A1219      -0.161  -4.706  -1.115                           H
HETATM  196 2H   HOH A1219      -1.519  -4.805  -1.767                           H
HETATM  197 1H   HOH A1220      -3.119   1.156   1.159                           H
HETATM  198 2H   HOH A1220      -4.688   1.715   0.993                           H
HETATM  199 1H   HOH A1223       2.778  -6.220  -0.377                           H
HETATM  200 2H   HOH A1223       1.698  -6.095   0.783                           H
HETATM  201 1H   HOH A1282      -0.182  -2.874  -5.817                           H
HETATM  202 2H   HOH A1282      -0.723  -1.977  -4.745                           H
```

```
HETATM  203 1H   HOH A1296       3.833  -3.165  -1.516                          H
HETATM  204 1H   HOH A1297       0.636  -3.503   0.652                          H
HETATM  205 1H   HOH A1298      -0.401  -2.439  -2.546                          H
HETATM  206  O   HOH A1300      -7.887  -1.095   3.185                          O
HETATM  207 1H   HOH A1300      -8.819  -0.861   3.278                          H
HETATM  208 2H   HOH A1300      -7.825  -1.333   2.237                          H
HETATM  209 2H   HOH A1297       1.724  -4.417  -0.024                          H
HETATM  210 2H   HOH A1298       0.849  -2.822  -3.390                          H
HETATM  211 2H   HOH A1296       3.014  -3.376  -2.840                          H
ATOM    212 3HD  ARG A 124       8.614   6.501  -0.690                          H
ATOM    213 3HA  GLY A 313       6.647  -1.968   5.332                          H
ATOM    214 3HA  GLU A 314       2.416  -3.788   6.334                          H
ATOM    215 2HA  ARG A 403      -6.631  -5.287   4.468                          H
ATOM    216 3HA  ARG A 403      -5.952  -5.997   2.988                          H
ATOM    217 3HG  ARG A 126       3.202   5.366  -3.780                          H
HETATM  218 1H5P ADP A 400       6.905  -0.867   0.143                          H
ATOM    219 3HG  ARG A 229       2.047   1.026  -7.413                          H
ATOM    220 3HG  ARG A 330      -5.069   8.133  -0.292                          H
ATOM    221 2HB  THR A 273      -6.838   2.778   2.471                          H
ATOM    222 3HB  THR A 273      -8.185   2.336   1.378                          H
ATOM    223 3HB  CYS A 271      -7.816  -0.902  -2.324                          H
ATOM    224 3HB  ASN A 274      -6.392   2.656  -2.384                          H
ATOM    225 3HB  GLU A 224       1.328  -5.861  -6.538                          H
ATOM    226 3HG  ARG A 280       1.279   5.538   0.797                          H
ATOM    227 3HB  GLU A 225      -2.981  -4.721  -4.026                          H
ATOM    228 3HD  ARG A 309       2.034   0.514   6.646                          H
HETATM  229  P   PO3 A 405      -0.699  -0.073   0.208                          P
```

IRC-Derived Reactant Cluster Model (229 atoms, charge +1)

```
ATOM      1  CG  ARG A 124      -4.843   5.993  -6.110                          C
ATOM      2  CD  ARG A 124      -4.933   6.063  -4.589                          C
ATOM      3  NE  ARG A 124      -3.634   6.065  -3.922                          N
ATOM      4  CZ  ARG A 124      -2.954   4.962  -3.571                          C
ATOM      5  NH1 ARG A 124      -3.408   3.751  -3.903                          N
ATOM      6  NH2 ARG A 124      -1.823   5.058  -2.880                          N
ATOM      7  CG  ARG A 126      -0.298   2.910  -5.570                          C
ATOM      8  CD  ARG A 126       0.605   1.688  -5.531                          C
ATOM      9  NE  ARG A 126       0.135   0.734  -4.535                          N
ATOM     10  CZ  ARG A 126       0.726  -0.445  -4.347                          C
ATOM     11  NH1 ARG A 126       0.266  -1.308  -3.423                          N
ATOM     12  NH2 ARG A 126       1.726  -0.839  -5.150                          N
ATOM     13  CG  GLU A 224       4.605   5.737   3.712                          C
ATOM     14  CD  GLU A 224       3.568   5.003   2.888                          C
ATOM     15  OE1 GLU A 224       3.933   4.207   1.994                          O
ATOM     16  OE2 GLU A 224       2.346   5.246   3.151                          O
ATOM     17  CG  GLU A 225       6.215   0.251   2.205                          C
ATOM     18  CD  GLU A 225       4.897  -0.311   1.725                          C
ATOM     19  OE1 GLU A 225       4.903  -1.320   0.959                          O
ATOM     20  OE2 GLU A 225       3.838   0.273   2.084                          O
ATOM     21  CG  ARG A 229       4.377   4.628  -2.564                          C
ATOM     22  CD  ARG A 229       4.595   3.364  -3.386                          C
ATOM     23  NE  ARG A 229       4.848   2.189  -2.563                          N
ATOM     24  CZ  ARG A 229       3.852   1.477  -2.001                          C
ATOM     25  NH1 ARG A 229       2.567   1.684  -2.330                          N
ATOM     26  NH2 ARG A 229       4.170   0.514  -1.145                          N
ATOM     27  CB  THR A 273       2.177  -7.031  -1.287                          C
ATOM     28  OG1 THR A 273       2.650  -5.695  -1.416                          O
ATOM     29  CB  ASN A 274       4.871  -3.651  -3.153                          C
ATOM     30  CG  ASN A 274       3.566  -3.001  -2.800                          C
ATOM     31  OD1 ASN A 274       2.617  -2.955  -3.630                          O
ATOM     32  ND2 ASN A 274       3.454  -2.495  -1.581                          N
```

ATOM	33	CG	ARG	A	280	-3.177	-1.404	-4.812	C
ATOM	34	CD	ARG	A	280	-3.118	-1.965	-3.396	C
ATOM	35	NE	ARG	A	280	-3.463	-0.978	-2.379	N
ATOM	36	CZ	ARG	A	280	-4.693	-0.537	-2.154	C
ATOM	37	NH1	ARG	A	280	-5.747	-1.140	-2.746	N
ATOM	38	NH2	ARG	A	280	-4.900	0.535	-1.396	N
ATOM	39	CD	ARG	A	309	-5.909	-1.637	0.909	C
ATOM	40	NE	ARG	A	309	-4.874	-2.529	0.400	N
ATOM	41	CZ	ARG	A	309	-3.544	-2.340	0.551	C
ATOM	42	NH1	ARG	A	309	-3.031	-1.152	0.771	N
ATOM	43	NH2	ARG	A	309	-2.729	-3.407	0.345	N
ATOM	44	CA	GLY	A	313	-6.643	2.408	3.290	C
ATOM	45	C	GLY	A	313	-6.671	1.061	3.986	C
ATOM	46	O	GLY	A	313	-7.536	0.738	4.778	O
ATOM	47	N	GLU	A	314	-5.632	0.229	3.666	N
ATOM	48	CA	GLU	A	314	-5.372	-0.938	4.496	C
ATOM	49	CB	GLU	A	314	-4.086	-1.634	4.070	C
ATOM	50	CG	GLU	A	314	-2.850	-0.750	4.184	C
ATOM	51	CD	GLU	A	314	-1.555	-1.398	3.703	C
ATOM	52	OE1	GLU	A	314	-1.622	-2.423	2.984	O
ATOM	53	OE2	GLU	A	314	-0.479	-0.816	4.039	O
ATOM	54	CG	ARG	A	330	0.960	-5.746	-6.529	C
ATOM	55	CD	ARG	A	330	0.823	-4.392	-5.838	C
ATOM	56	NE	ARG	A	330	0.395	-4.489	-4.447	N
ATOM	57	CZ	ARG	A	330	-0.859	-4.698	-4.058	C
ATOM	58	NH1	ARG	A	330	-1.840	-4.829	-5.008	N
ATOM	59	NH2	ARG	A	330	-1.160	-4.729	-2.774	N
HETATM	60	O1G	ATP	A	400	-0.226	0.314	1.136	O
HETATM	61	O2G	ATP	A	400	-0.402	-1.999	-0.085	O
HETATM	62	O3G	ATP	A	400	1.496	-0.377	-0.750	O
HETATM	63	MG	MG	A	408	0.268	2.271	1.433	Mg
ATOM	64	CA	ARG	A	403	1.581	-5.551	6.548	C
ATOM	65	CB	ARG	A	403	0.932	-5.086	5.248	C
ATOM	66	CG	ARG	A	403	1.738	-5.506	4.021	C
ATOM	67	CD	ARG	A	403	1.054	-5.170	2.696	C
ATOM	68	NE	ARG	A	403	0.841	-3.741	2.498	N
ATOM	69	CZ	ARG	A	403	1.822	-2.898	2.156	C
ATOM	70	NH1	ARG	A	403	2.969	-3.327	1.636	N
ATOM	71	NH2	ARG	A	403	1.613	-1.582	2.289	N
HETATM	72	PB	ATP	A	400	-1.258	1.758	-1.321	P
HETATM	73	O1B	ATP	A	400	-0.149	2.557	-0.636	O
HETATM	74	O2B	ATP	A	400	-1.570	2.093	-2.788	O
HETATM	75	O3B	ATP	A	400	-1.010	0.171	-1.275	O
HETATM	76	PA	ATP	A	400	-3.040	2.362	1.080	P
HETATM	77	O1A	ATP	A	400	-1.754	2.615	1.823	O
HETATM	78	O2A	ATP	A	400	-4.042	1.272	1.427	O
HETATM	79	O3A	ATP	A	400	-2.679	2.006	-0.543	O
HETATM	80	O5P	ATP	A	400	-3.889	3.721	0.891	O
HETATM	81	C5P	ADP	A	400	-3.147	4.952	0.858	C
ATOM	82	CB	CYS	A	271	5.912	-4.864	1.154	C
ATOM	83	SG	CYS	A	271	4.492	-5.991	0.896	S
HETATM	84	O	HOH	A	1003	0.858	5.456	5.296	O
HETATM	85	O	HOH	A	1210	0.966	4.056	-2.592	O
HETATM	86	O	HOH	A	1212	6.397	-1.333	-1.136	O
HETATM	87	O	HOH	A	1214	-0.088	5.766	-0.649	O
HETATM	88	O	HOH	A	1215	-1.296	4.919	3.648	O
HETATM	89	O	HOH	A	1216	0.562	0.599	6.236	O
HETATM	90	O	HOH	A	1219	2.851	1.087	4.653	O
HETATM	91	O	HOH	A	1220	0.804	-3.938	-1.432	O
HETATM	92	O	HOH	A	1223	-0.397	2.978	5.480	O
HETATM	93	O	HOH	A	1282	5.450	2.825	0.179	O
HETATM	94	O	HOH	A	1296	0.419	4.315	1.693	O
HETATM	95	O	HOH	A	1297	0.439	1.715	3.371	O

```
HETATM   96  O   HOH A1298       2.297   2.262   1.079                       O
ATOM     97 1HG  ARG A 124      -4.331   5.080  -6.447                       H
ATOM     98 2HG  ARG A 124      -4.288   6.854  -6.508                       H
ATOM     99 1HD  ARG A 124      -5.516   5.225  -4.178                       H
ATOM    100 2HD  ARG A 124      -5.454   6.978  -4.278                       H
ATOM    101  HE  ARG A 124      -3.162   6.951  -3.784                       H
ATOM    102 1HH1 ARG A 124      -4.081   3.650  -4.649                       H
ATOM    103 2HH1 ARG A 124      -2.871   2.943  -3.537                       H
ATOM    104 1HH2 ARG A 124      -1.240   4.226  -2.757                       H
ATOM    105 2HH2 ARG A 124      -1.574   5.851  -2.295                       H
ATOM    106 1HG  ARG A 126      -1.343   2.609  -5.737                       H
ATOM    107 2HG  ARG A 126      -0.220   3.466  -4.626                       H
ATOM    108 1HD  ARG A 126       0.621   1.191  -6.515                       H
ATOM    109 2HD  ARG A 126       1.638   1.998  -5.287                       H
ATOM    110  HE  ARG A 126      -0.538   1.087  -3.834                       H
ATOM    111 1HH1 ARG A 126      -0.226  -0.870  -2.642                       H
ATOM    112 2HH1 ARG A 126       0.942  -2.033  -3.143                       H
ATOM    113 1HH2 ARG A 126       2.278  -1.634  -4.796                       H
ATOM    114 2HH2 ARG A 126       2.206  -0.134  -5.694                       H
ATOM    115 1HB  GLU A 224       4.388   6.814   3.699                       H
ATOM    116 2HB  GLU A 224       4.531   5.394   4.754                       H
ATOM    117 1HG  GLU A 225       6.473   1.093   1.543                       H
ATOM    118 2HG  GLU A 225       7.007  -0.507   2.168                       H
ATOM    119 1HG  ARG A 229       3.502   4.520  -1.906                       H
ATOM    120 2HG  ARG A 229       5.250   4.825  -1.927                       H
ATOM    121 1HD  ARG A 229       3.729   3.151  -4.029                       H
ATOM    122 2HD  ARG A 229       5.460   3.482  -4.055                       H
ATOM    123  HE  ARG A 229       5.645   2.276  -1.931                       H
ATOM    124 1HH2 ARG A 229       3.411   0.017  -0.672                       H
ATOM    125 2HH2 ARG A 229       5.100   0.085  -1.168                       H
ATOM    126 1HH1 ARG A 229       1.915   1.129  -1.756                       H
ATOM    127 2HH1 ARG A 229       2.277   2.640  -2.551                       H
ATOM    128 1HB  THR A 273       2.120  -7.338  -0.229                       H
ATOM    129  HG1 THR A 273       3.403  -5.614  -0.733                       H
ATOM    130 1HB  ASN A 274       5.700  -3.169  -2.619                       H
ATOM    131 2HB  ASN A 274       5.020  -3.602  -4.238                       H
ATOM    132 1HD2 ASN A 274       2.591  -2.033  -1.278                       H
ATOM    133 2HD2 ASN A 274       4.240  -2.506  -0.935                       H
ATOM    134 1HG  ARG A 280      -2.903  -2.189  -5.530                       H
ATOM    135 2HG  ARG A 280      -4.183  -1.034  -5.058                       H
ATOM    136 1HD  ARG A 280      -3.766  -2.853  -3.283                       H
ATOM    137 2HD  ARG A 280      -2.101  -2.300  -3.157                       H
ATOM    138  HE  ARG A 280      -2.696  -0.595  -1.809                       H
ATOM    139 1HH2 ARG A 280      -5.804   0.705  -0.973                       H
ATOM    140 2HH2 ARG A 280      -4.100   1.055  -1.013                       H
ATOM    141 1HH1 ARG A 280      -6.681  -0.801  -2.562                       H
ATOM    142 2HH1 ARG A 280      -5.651  -2.081  -3.100                       H
ATOM    143 1HD  ARG A 309      -6.714  -1.554   0.164                       H
ATOM    144 2HD  ARG A 309      -5.498  -0.639   1.107                       H
ATOM    145  HE  ARG A 309      -5.142  -3.495   0.253                       H
ATOM    146 1HH1 ARG A 309      -3.574  -0.295   0.984                       H
ATOM    147 2HH1 ARG A 309      -2.054  -1.147   1.082                       H
ATOM    148 1HH2 ARG A 309      -3.005  -4.240   0.858                       H
ATOM    149 2HH2 ARG A 309      -1.738  -3.153   0.483                       H
ATOM    150 1HA  GLY A 313      -6.254   2.334   2.266                       H
ATOM    151 2HA  GLY A 313      -5.972   3.078   3.849                       H
ATOM    152 1HB  GLU A 314      -3.945  -2.531   4.692                       H
ATOM    153 2HB  GLU A 314      -4.179  -2.000   3.036                       H
ATOM    154 1HG  GLU A 314      -2.700  -0.414   5.221                       H
ATOM    155 2HG  GLU A 314      -2.947   0.175   3.586                       H
ATOM    156 1HA  GLU A 314      -6.231  -1.623   4.423                       H
ATOM    157 1HA  GLU A 314      -4.859   0.646   3.154                       H
ATOM    158 1HG  ARG A 330       0.008  -6.298  -6.580                       H
```

```
ATOM    159 2HG  ARG A 330       1.677  -6.377  -5.988                           H
ATOM    160 1HD  ARG A 330       0.137  -3.723  -6.386                           H
ATOM    161 2HD  ARG A 330       1.797  -3.889  -5.801                           H
ATOM    162  HE  ARG A 330       1.089  -4.248  -3.728                           H
ATOM    163 1HH1 ARG A 330      -2.663  -5.340  -4.706                           H
ATOM    164 2HH1 ARG A 330      -1.532  -5.099  -5.936                           H
ATOM    165 1HH2 ARG A 330      -2.126  -4.850  -2.499                           H
ATOM    166 2HH2 ARG A 330      -0.358  -4.437  -2.055                           H
ATOM    167 1HB  ARG A 403      -0.085  -5.510   5.172                           H
ATOM    168 2HB  ARG A 403       0.811  -3.989   5.260                           H
ATOM    169 1HG  ARG A 403       1.905  -6.594   4.032                           H
ATOM    170 2HG  ARG A 403       2.735  -5.032   4.039                           H
ATOM    171 1HD  ARG A 403       0.064  -5.647   2.657                           H
ATOM    172 2HD  ARG A 403       1.632  -5.581   1.854                           H
ATOM    173  HE  ARG A 403      -0.034  -3.298   2.812                           H
ATOM    174 1HH1 ARG A 403       3.221  -4.327   1.505                           H
ATOM    175 2HH1 ARG A 403       3.682  -2.624   1.430                           H
ATOM    176 1HH2 ARG A 403       0.848  -1.275   2.902                           H
ATOM    177 2HH2 ARG A 403       2.393  -0.944   2.126                           H
ATOM    178 1HA  ARG A 403       0.994  -5.247   7.426                           H
ATOM    179 1HB  CYS A 271       5.585  -3.833   1.362                           H
ATOM    180 2HB  CYS A 271       6.508  -5.208   2.010                           H
HETATM  181 1H5P ATP A 400      -2.392   4.932   0.057                           H
HETATM  182 2H5P ATP A 400      -2.654   5.128   1.823                           H
HETATM  183 1H   HOH A1003       1.132   6.146   5.911                           H
HETATM  184 2H   HOH A1003       1.544   5.440   4.575                           H
HETATM  185 1H   HOH A1210       0.627   3.501  -1.853                           H
HETATM  186 2H   HOH A1210       0.974   4.925  -2.153                           H
HETATM  187 1H   HOH A1212       6.039  -1.449  -0.217                           H
HETATM  188 2H   HOH A1212       7.356  -1.386  -1.043                           H
HETATM  189 1H   HOH A1214       0.199   6.594  -0.238                           H
HETATM  190 2H   HOH A1214       0.037   5.121   0.091                           H
HETATM  191 1H   HOH A1215      -0.693   5.326   4.305                           H
HETATM  192 2H   HOH A1215      -1.480   4.053   4.055                           H
HETATM  193 1H   HOH A1216       1.482   0.584   5.905                           H
HETATM  194 2H   HOH A1216       0.107  -0.028   5.641                           H
HETATM  195 1H   HOH A1219       2.132   1.408   4.079                           H
HETATM  196 2H   HOH A1219       3.465   0.702   4.008                           H
HETATM  197 1H   HOH A1220       0.600  -3.302  -0.707                           H
HETATM  198 2H   HOH A1220       1.568  -4.578  -1.234                           H
HETATM  199 1H   HOH A1223       0.206   3.710   5.707                           H
HETATM  200 2H   HOH A1223      -0.088   2.172   5.974                           H
HETATM  201 1H   HOH A1282       5.295   3.531   0.835                           H
HETATM  202 2H   HOH A1282       4.724   2.213   0.392                           H
HETATM  203 1H   HOH A1296      -0.302   4.544   2.347                           H
HETATM  204 1H   HOH A1297       0.008   0.837   3.511                           H
HETATM  205 1H   HOH A1298       2.775   1.469   1.413                           H
HETATM  206  O   HOH A1300       2.365  -7.956   2.147                           O
HETATM  207 1H   HOH A1300       2.808  -8.782   1.915                           H
HETATM  208 2H   HOH A1300       3.103  -7.313   2.111                           H
HETATM  209 2H   HOH A1297       0.106   2.284   4.129                           H
HETATM  210 2H   HOH A1298       2.818   3.031   1.445                           H
HETATM  211 2H   HOH A1296       1.253   4.640   2.176                           H
ATOM    212 3HD  ARG A 124      -5.851   6.004  -6.548                           H
ATOM    213 3HA  GLY A 313      -7.651   2.836   3.304                           H
ATOM    214 3HA  GLU A 314      -5.316  -0.638   5.557                           H
ATOM    215 2HA  ARG A 403       1.679  -6.647   6.565                           H
ATOM    216 3HA  ARG A 403       2.591  -5.126   6.654                           H
ATOM    217 3HG  ARG A 126       0.012   3.567  -6.393                           H
HETATM  218 1H5P ATP A 400      -3.882   5.740   0.656                           H
ATOM    219 3HG  ARG A 229       4.217   5.490  -3.226                           H
ATOM    220 3HG  ARG A 330       1.322  -5.609  -7.558                           H
ATOM    221 2HB  THR A 273       1.171  -7.085  -1.732                           H
```

```
ATOM    222 3HB  THR A 273       2.838  -7.730  -1.823                           H
ATOM    223 3HB  CYS A 271       6.561  -4.851   0.267                           H
ATOM    224 3HB  ASN A 274       4.804  -4.706  -2.847                           H
ATOM    225 3HB  GLU A 224       5.616   5.546   3.341                           H
ATOM    226 3HG  ARG A 280      -2.462  -0.574  -4.911                           H
ATOM    227 3HB  GLU A 225       6.108   0.652   3.220                           H
ATOM    228 3HD  ARG A 309      -6.334  -2.008   1.853                           H
HETATM  229  PG  ATP A 400       0.151  -0.575  -0.052                           P
```

Large TS Cluster Model (275 atoms, charge -1)

Mg	25.79	10.63	36.945
O	27.828	10.683	37.318
O	25.873	12.084	35.517
H	28.118	10.269	38.182
H	28.418	10.296	36.656
O	25.984	9.288	35.392
O	23.731	10.383	36.84
O	25.714	9.317	38.512
O	25.45	12.205	38.256
H	26.172	12.915	35.907
H	26.188	12.099	34.536
H	26.087	9.65	34.467
H	26.616	8.535	35.44
P	22.85	10.24	38.049
P	25.071	7.99	38.775
P	24.433	12.45	39.323
O	23.113	11.525	39.046
O	21.35	10.341	37.755
O	23.119	9.004	38.935
O	24.675	7.075	37.667
O	24.951	7.512	40.196
O	23.781	13.908	39.194
O	24.785	12.312	40.775
C	23.167	14.284	37.96
H	22.999	15.366	38
H	23.822	14.042	37.11
H	22.206	13.763	37.844
C	26.618	11.219	32.383
C	27.02	11.374	30.913
O	26.332	10.075	32.82
O	26.614	12.277	33.083
H	26.534	12.259	30.483

H	26.769	10.476	30.337
H	28.107	11.536	30.869
N	24.739	6.288	34.847
C	23.491	6.772	34.709
H	24.883	5.318	34.569
H	25.27	6.648	35.641
N	23.203	7.971	35.198
N	22.584	6.036	34.06
H	22.281	8.396	35.104
H	23.933	8.5	35.68
C	21.276	6.553	33.692
H	22.952	5.155	33.652
C	21.315	7.517	32.511
H	20.835	7.05	34.572
H	20.644	5.686	33.462
H	21.964	8.377	32.729
H	20.305	7.893	32.293
H	21.703	7.013	31.615
C	28.164	9.688	40.681
C	28.073	11.163	41.053
O	28.605	9.431	39.518
O	27.802	8.817	41.51
C	28.038	11.427	42.552
H	27.165	11.547	40.554
H	28.924	11.681	40.585
C	28.065	12.905	42.911
H	28.908	10.947	43.024
H	27.152	10.945	42.991
N	26.911	13.611	42.377
H	28.086	13.036	44.005
H	28.988	13.373	42.528
C	26.77	14.939	42.6
H	26.269	13.142	41.736
C	25.602	15.606	41.905
O	27.545	15.566	43.315
H	26.001	16.329	41.18
H	24.955	14.89	41.381
H	25.024	16.169	42.649
N	24.846	9.775	41.85
C	25.144	9.254	43.023

H	24.827	10.785	41.638
H	24.937	9.124	41.058
N	25.088	9.952	44.163
N	25.426	7.915	43.077
C	24.771	11.368	44.277
H	25.428	9.487	44.997
H	25.975	7.637	43.887
H	25.863	7.605	42.201
H	24.102	11.523	45.131
H	24.272	11.709	43.36
H	25.681	11.971	44.409
C	27.579	6.025	35.057
C	27.875	5.988	33.575
O	27.609	7.148	35.649
O	27.321	4.948	35.653
H	27.391	5.125	33.1
H	27.564	6.925	33.097
H	28.964	5.884	33.451
N	22.046	6.398	38.799
C	21.066	6.332	37.9
H	22.507	7.311	38.889
H	22.631	5.56	38.856
N	20.551	7.457	37.402
N	20.569	5.128	37.563
C	19.183	7.599	36.916
H	20.987	8.329	37.717
H	21.122	4.325	37.865
H	20.067	5.039	36.688
C	18.527	8.736	37.679
H	18.647	6.656	37.09
H	19.185	7.814	35.839
H	18.571	8.551	38.763
H	19.041	9.681	37.46
H	17.475	8.839	37.382
N	27.387	7.176	38.515
C	27.816	6.054	39.156
H	27.823	8.045	38.875
H	27.391	7.108	37.49
N	28.109	6.167	40.452
N	27.873	4.909	38.496

C	28.857	5.156	41.191
H	28.063	7.129	40.832
H	27.669	4.925	37.488
H	28.194	4.012	38.918
C	30.326	5.073	40.774
H	28.778	5.432	42.252
H	28.371	4.175	41.065
C	31.017	6.437	40.765
H	30.835	4.382	41.468
H	30.396	4.609	39.777
C	32.538	6.338	40.601
H	30.611	7.043	39.938
H	30.811	6.982	41.7
N	32.876	5.49	39.454
C	33.221	5.885	41.912
H	32.919	7.352	40.403
H	33.895	5.467	39.373
H	32.622	4.513	39.67
H	32.057	5.591	38.039
O	33.877	4.808	41.888
O	33.087	6.637	42.91
C	31.979	4.376	36.516
C	33.318	3.97	36.629
C	31.13	3.644	35.677
O	31.491	5.445	37.194
C	33.773	2.837	35.954
H	34.002	4.541	37.257
C	31.606	2.523	34.995
H	30.086	3.955	35.584
C	32.932	2.085	35.126
H	34.815	2.532	36.079
H	30.924	1.948	34.366
C	33.433	0.858	34.396
H	33.941	1.132	33.458
H	32.591	0.197	34.146
H	34.152	0.299	35.012
N	33.955	3.233	44.246
C	33.863	1.879	43.75
C	34.004	3.511	45.566
H	33.968	3.998	43.561

C	35.016	1.462	42.83
H	32.926	1.731	43.186
H	33.862	1.197	44.606
C	34.125	5.001	45.9
O	33.916	2.673	46.457
N	35.519	2.429	42.029
O	35.424	0.306	42.826
N	34.883	5.774	44.946
H	33.109	5.417	45.961
H	34.593	5.057	46.89
C	36.549	2.131	41.053
H	35.031	3.322	41.96
C	36.235	5.67	44.95
H	34.372	6.2	44.159
H	37.066	3.06	40.784
H	36.138	1.675	40.138
H	37.264	1.426	41.494
C	36.967	6.423	43.859
O	36.839	5.014	45.793
H	37.766	7.021	44.316
H	36.303	7.066	43.269
H	37.437	5.685	43.192
N	19.323	11.854	39.165
C	19.091	12.695	38.147
H	20.025	11.127	38.989
H	18.594	11.667	39.842
N	19.872	12.597	37.072
N	18.132	13.621	38.206
H	20.596	11.864	37.085
H	19.867	13.312	36.357
C	17.28	13.912	39.358
H	17.993	14.18	37.371
C	15.872	13.342	39.224
H	17.244	15.005	39.459
H	17.786	13.54	40.258
H	15.276	13.619	40.103
H	15.376	13.742	38.329
H	15.895	12.245	39.146
C	24.434	3.555	32.098
C	23.78	4.36	30.986

O	25.282	2.693	31.802
O	24.067	3.828	33.293
H	22.686	4.292	31.072
H	24.1	4.009	30
H	24.054	5.42	31.104
C	23.762	3.362	37.749
C	23.71	1.932	37.282
N	24.654	4.167	37.2
O	22.964	3.751	38.651
C	23.542	0.993	38.469
H	24.604	1.704	36.688
H	22.842	1.853	36.606
H	25.271	3.784	36.475
H	24.711	5.163	37.455
H	24.378	1.104	39.176
H	22.603	1.202	39.001
H	23.52	-0.051	38.132
N	26.715	0.408	32.728
C	28.155	0.366	32.67
C	26.026	-0.731	32.982
H	26.21	1.276	32.524
C	28.868	0.269	34.022
H	28.513	1.277	32.169
H	28.491	-0.507	32.094
C	24.517	-0.609	32.93
O	26.586	-1.796	33.221
N	28.24	0.847	35.061
O	29.975	-0.262	34.096
H	24.188	0.426	32.766
H	24.101	-0.993	33.871
H	24.145	-1.247	32.116
C	28.782	0.817	36.413
H	27.315	1.256	34.916
C	28.185	1.936	37.256
H	28.568	-0.156	36.888
H	29.875	0.916	36.354
H	28.445	2.901	36.79
H	27.086	1.855	37.228
S	28.749	1.897	39.005
N	21.664	10.362	43.585

C	21.804	10.408	42.255
H	21.545	11.213	44.119
H	21.613	9.481	44.077
N	21.928	11.599	41.656
N	21.769	9.293	41.529
H	22.26	12.385	42.205
H	22.233	11.602	40.679
C	21.528	7.982	42.128
H	22.211	9.307	40.596
C	20.079	7.799	42.579
H	21.783	7.246	41.354
H	22.237	7.815	42.957
H	19.403	7.891	41.719
H	19.778	8.54	43.331
H	19.955	6.799	43.015
N	23.079	5.135	43.102
C	23.153	4.47	41.932
H	22.672	4.676	43.908
H	23.803	5.824	43.291
N	23.957	4.923	40.974
N	22.415	3.37	41.738
H	24.352	5.874	40.969
H	23.885	4.46	40.064
C	21.542	2.785	42.764
H	22.416	2.998	40.788
C	20.85	1.5	42.302
H	22.152	2.566	43.655
H	20.782	3.531	43.051
H	21.59	0.732	42.04
H	20.225	1.122	43.119
H	20.209	1.691	41.43
O	31.925	2.624	39.25
H	32.235	2.494	38.341
H	30.96	2.443	39.178
O	25.947	2.724	34.997
H	26.618	3.441	35.014
H	25.306	2.993	34.298
O	20.799	9.618	35.159
H	21.142	10.3	34.564
H	21.052	9.936	36.054

O	30.158	7.961	36.52
H	29.282	7.717	36.175
H	30.572	7.1	36.715

Large Reactant Cluster Model (275 atoms, charge -1)

Mg	25.572	10.846	36.875
O	27.563	10.955	37.262
O	25.634	12.361	35.485
H	27.757	10.596	38.168
H	28.135	10.352	36.735
O	25.759	9.574	35.272
O	23.42	10.883	36.792
O	25.316	9.286	38.151
O	25.351	12.319	38.337
H	26.13	13.096	35.872
H	26	12.268	34.536
H	25.883	9.876	34.311
H	26.316	8.765	35.354
P	22.658	10.476	38.026
P	24.363	8.192	38.579
P	24.36	12.447	39.447
O	23.28	11.212	39.323
O	21.173	10.73	38.052
O	22.833	8.944	38.38
O	24.241	6.987	37.674
O	24.367	7.864	40.065
O	23.435	13.742	39.26
O	24.762	12.439	40.886
C	23.167	14.284	37.96
H	22.734	15.277	38.116
H	24.094	14.36	37.378
H	22.451	13.646	37.419
C	26.5	11.271	32.349
C	27.02	11.374	30.913
O	26.075	10.15	32.747
O	26.547	12.312	33.064
H	26.84	12.377	30.509
H	26.563	10.607	30.277
H	28.107	11.204	30.934

N	24.288	6.413	34.809
C	23.222	7.171	34.529
H	24.253	5.434	34.512
H	24.838	6.661	35.632
N	23.155	8.41	35.029
N	22.237	6.67	33.774
H	22.417	9.045	34.732
H	24.033	8.845	35.337
C	21.233	7.507	33.131
H	22.43	5.717	33.413
C	21.78	8.292	31.943
H	20.796	8.186	33.88
H	20.424	6.837	32.812
H	22.593	8.964	32.256
H	20.985	8.898	31.486
H	22.179	7.607	31.181
C	28.012	9.757	40.588
C	28.065	11.226	40.992
O	28.311	9.511	39.379
O	27.666	8.892	41.428
C	28.069	11.47	42.494
H	27.19	11.7	40.511
H	28.952	11.674	40.519
C	28.126	12.945	42.864
H	28.938	10.965	42.943
H	27.18	10.999	42.94
N	26.957	13.651	42.366
H	28.18	13.069	43.958
H	29.038	13.408	42.452
C	26.807	14.979	42.575
H	26.286	13.171	41.764
C	25.602	15.606	41.905
O	27.599	15.632	43.246
H	25.954	16.214	41.059
H	24.892	14.856	41.535
H	25.113	16.282	42.618
N	24.352	9.891	41.901
C	24.938	9.309	42.936
H	24.435	10.907	41.747
H	24.292	9.286	41.058

N	25.156	9.976	44.071
N	25.205	7.973	42.859
C	24.771	11.368	44.277
H	25.702	9.515	44.789
H	25.899	7.642	43.525
H	25.441	7.704	41.896
H	24.802	11.578	45.35
H	23.747	11.517	43.91
H	25.444	12.062	43.75
C	27.328	6.146	34.981
C	27.875	5.988	33.575
O	27.245	7.321	35.455
O	26.986	5.127	35.634
H	27.732	4.967	33.203
H	27.39	6.715	32.909
H	28.95	6.221	33.595
N	21.774	6.032	37.088
C	20.633	6.569	36.7
H	22.637	6.589	37.266
H	21.818	5.051	37.372
N	20.539	7.891	36.517
N	19.551	5.783	36.512
C	19.284	8.611	36.363
H	21.332	8.423	36.871
H	19.718	4.792	36.381
H	18.761	6.16	36.004
C	18.527	8.736	37.679
H	18.675	8.118	35.589
H	19.55	9.596	35.961
H	18.273	7.747	38.085
H	19.152	9.272	38.408
H	17.595	9.301	37.533
N	27.289	7.092	38.377
C	27.763	6.045	39.054
H	27.425	8.031	38.758
H	27.044	6.997	37.393
N	28.106	6.223	40.338
N	27.832	4.857	38.46
C	28.864	5.245	41.11
H	28.032	7.187	40.694

H	27.576	4.821	37.472
H	28.22	4.007	38.907
C	30.34	5.16	40.723
H	28.767	5.544	42.163
H	28.393	4.255	41.005
C	31.046	6.515	40.766
H	30.828	4.446	41.41
H	30.429	4.722	39.716
C	32.571	6.402	40.627
H	30.664	7.146	39.946
H	30.826	7.037	41.711
N	32.916	5.569	39.474
C	33.229	5.924	41.943
H	32.965	7.415	40.453
H	33.935	5.563	39.388
H	32.678	4.589	39.693
H	32.035	5.599	38.023
O	33.868	4.837	41.918
O	33.097	6.671	42.947
C	31.954	4.338	36.556
C	33.276	3.892	36.726
C	31.138	3.655	35.644
O	31.449	5.396	37.223
C	33.739	2.771	36.037
H	33.94	4.421	37.41
C	31.619	2.542	34.956
H	30.114	4.005	35.496
C	32.924	2.068	35.143
H	34.765	2.435	36.206
H	30.959	2.004	34.273
C	33.433	0.858	34.396
H	33.961	1.148	33.474
H	32.594	0.206	34.114
H	34.136	0.279	35.011
N	33.957	3.23	44.252
C	33.863	1.879	43.75
C	34.022	3.5	45.574
H	33.96	4	43.572
C	35.013	1.468	42.824
H	32.925	1.734	43.188

H	33.865	1.192	44.602
C	34.143	4.988	45.915
O	33.948	2.656	46.46
N	35.519	2.437	42.028
O	35.419	0.31	42.81
N	34.893	5.768	44.961
H	33.127	5.402	45.987
H	34.618	5.038	46.903
C	36.549	2.131	41.053
H	35.04	3.336	41.967
C	36.245	5.663	44.951
H	34.375	6.203	44.183
H	37.067	3.057	40.775
H	36.132	1.668	40.144
H	37.266	1.429	41.495
C	36.967	6.423	43.859
O	36.856	4.999	45.783
H	37.779	7.006	44.311
H	36.3	7.08	43.288
H	37.419	5.69	43.175
N	19.147	12.43	38.679
C	18.623	12.739	37.494
H	19.942	11.774	38.674
H	18.654	12.632	39.537
N	19.177	12.222	36.391
N	17.569	13.555	37.398
H	20.044	11.681	36.458
H	18.952	12.616	35.486
C	16.923	14.207	38.535
H	17.172	13.694	36.477
C	15.872	13.342	39.224
H	16.469	15.128	38.15
H	17.709	14.523	39.237
H	15.426	13.895	40.061
H	15.075	13.071	38.519
H	16.309	12.412	39.618
C	24.003	3.742	32.358
C	23.78	4.36	30.986
O	24.881	2.851	32.475
O	23.31	4.188	33.32

H	22.71	4.537	30.813
H	24.199	3.733	30.192
H	24.288	5.337	30.968
C	23.54	3.302	37.611
C	23.617	1.853	37.212
N	24.474	4.123	37.158
O	22.607	3.681	38.372
C	23.542	0.993	38.469
H	24.53	1.67	36.633
H	22.754	1.648	36.558
H	25.145	3.764	36.471
H	24.488	5.115	37.424
H	24.362	1.239	39.16
H	22.586	1.151	38.987
H	23.622	-0.072	38.215
N	26.658	0.531	32.723
C	28.101	0.561	32.656
C	26.031	-0.648	32.964
H	26.108	1.363	32.52
C	28.826	0.373	33.994
H	28.411	1.529	32.239
H	28.474	-0.24	32.004
C	24.517	-0.609	32.93
O	26.654	-1.68	33.183
N	28.217	0.897	35.071
O	29.925	-0.179	34.019
H	24.128	0.409	32.8
H	24.137	-1.04	33.865
H	24.172	-1.246	32.104
C	28.782	0.817	36.413
H	27.311	1.358	34.958
C	28.286	1.974	37.271
H	28.503	-0.139	36.889
H	29.878	0.836	36.33
H	28.624	2.916	36.809
H	27.183	1.983	37.253
S	28.865	1.879	39.012
N	21.242	10.779	43.193
C	21.223	10.734	41.86
H	21.207	11.667	43.677

H	21.218	9.935	43.749
N	21.141	11.887	41.159
N	21.23	9.57	41.224
H	21.36	12.74	41.666
H	21.571	11.854	40.234
C	21.364	8.278	41.9
H	21.247	9.595	40.204
C	20.079	7.799	42.579
H	21.684	7.569	41.127
H	22.2	8.352	42.616
H	19.277	7.679	41.839
H	19.724	8.495	43.352
H	20.27	6.827	43.053
N	22.919	5.272	42.765
C	22.723	4.699	41.552
H	22.985	4.662	43.572
H	23.547	6.076	42.792
N	23.049	5.368	40.456
N	22.127	3.499	41.473
H	23.587	6.251	40.472
H	22.913	4.891	39.548
C	21.759	2.688	42.642
H	22.086	3.094	40.536
C	20.85	1.5	42.302
H	22.674	2.317	43.133
H	21.238	3.346	43.354
H	21.351	0.808	41.611
H	20.62	0.959	43.227
H	19.911	1.842	41.847
O	32.004	2.675	39.401
H	32.359	2.496	38.517
H	31.042	2.504	39.28
O	25.925	2.751	35.01
H	26.493	3.553	35.035
H	25.44	2.808	34.152
O	21.422	10.84	34.95
H	21.621	11.316	34.131
H	22.208	11.001	35.521
O	29.209	8.917	36.563
H	28.634	8.243	36.146

H	29.192	8.693	37.508

Large NPC Cluster Model (275 atoms, charge -1)

Mg	25.577	10.52	36.975
O	27.655	10.71	37.156
O	25.594	12.006	35.52
H	28.023	10.378	38.013
H	28.165	10.256	36.47
O	25.846	9.22	35.378
O	23.554	10.358	36.999
O	25.625	9.072	38.396
O	25.431	12.078	38.318
H	25.994	12.781	35.937
H	25.932	12.016	34.557
H	25.974	9.585	34.46
H	26.499	8.489	35.442
P	22.533	10.538	38.092
P	25.739	7.617	38.774
P	24.433	12.509	39.347
O	23.026	11.743	39.112
O	21.183	11.026	37.558
O	22.335	9.369	39.055
O	25.179	6.611	37.794
O	25.454	7.313	40.229
O	23.961	14.023	39.118
O	24.776	12.406	40.806
C	23.167	14.284	37.96
H	23.164	15.369	37.808
H	23.595	13.793	37.072
H	22.141	13.923	38.117
C	26.559	11.175	32.367
C	27.02	11.374	30.913
O	26.343	10.008	32.789
O	26.425	12.223	33.067
H	27.041	10.423	30.369
H	28.025	11.82	30.92
H	26.347	12.083	30.413
N	24.698	6.151	35.066
C	23.466	6.662	34.912

H	24.859	5.232	34.654
H	25.175	6.384	35.943
N	23.125	7.759	35.591
N	22.614	6.049	34.086
H	22.259	8.271	35.41
H	23.864	8.284	36.061
C	21.348	6.635	33.683
H	22.993	5.204	33.625
C	21.505	7.73	32.632
H	20.841	7.037	34.574
H	20.723	5.818	33.3
H	22.182	8.519	32.992
H	20.53	8.184	32.404
H	21.928	7.317	31.705
C	28.231	9.768	40.61
C	28.12	11.229	40.998
O	28.566	9.536	39.393
O	27.99	8.871	41.443
C	28.091	11.465	42.502
H	27.203	11.603	40.505
H	28.958	11.767	40.53
C	28.106	12.937	42.883
H	28.966	10.982	42.96
H	27.209	10.97	42.938
N	26.94	13.633	42.366
H	28.137	13.051	43.978
H	29.019	13.422	42.497
C	26.795	14.963	42.577
H	26.277	13.156	41.753
C	25.602	15.606	41.905
O	27.585	15.604	43.262
H	25.972	16.322	41.157
H	24.95	14.875	41.411
H	25.039	16.176	42.656
N	24.929	9.834	41.749
C	25.174	9.284	42.927
H	24.835	10.847	41.575
H	25.109	9.235	40.934
N	25.072	9.953	44.086
N	25.456	7.956	42.965

C	24.771	11.368	44.277
H	25.322	9.43	44.917
H	26.004	7.645	43.761
H	25.784	7.601	42.054
H	24.058	11.475	45.102
H	24.326	11.777	43.359
H	25.683	11.941	44.498
C	27.649	6.077	35.072
C	27.875	5.989	33.574
O	27.731	7.207	35.648
O	27.415	5.007	35.693
H	27.23	5.216	33.135
H	27.704	6.957	33.091
H	28.921	5.689	33.411
N	22.509	6.631	38.675
C	21.433	6.517	37.88
H	22.726	7.608	38.911
H	23.331	6.102	38.379
N	20.59	7.542	37.806
N	21.204	5.376	37.23
C	19.286	7.527	37.175
H	20.931	8.405	38.246
H	21.805	4.579	37.455
H	20.402	5.27	36.625
C	18.527	8.736	37.679
H	18.762	6.595	37.441
H	19.383	7.579	36.079
H	18.422	8.697	38.774
H	19.068	9.655	37.409
H	17.525	8.778	37.233
N	27.533	7.37	38.525
C	28.101	6.204	39.111
H	28.066	8.289	38.903
H	27.648	7.316	37.488
N	28.478	6.331	40.365
N	28.177	5.116	38.39
C	29.201	5.294	41.096
H	28.332	7.265	40.8
H	27.849	5.135	37.404
H	28.554	4.201	38.756

C	30.666	5.197	40.672
H	29.127	5.562	42.157
H	28.694	4.328	40.949
C	31.372	6.554	40.664
H	31.164	4.499	41.366
H	30.734	4.734	39.674
C	32.899	6.425	40.605
H	31.026	7.138	39.795
H	31.119	7.129	41.569
N	33.292	5.531	39.511
C	33.47	5.989	41.972
H	33.316	7.423	40.404
H	34.313	5.501	39.474
H	33.018	4.566	39.75
H	32.492	5.628	38.127
O	34.091	4.892	42.016
O	33.282	6.764	42.943
C	32.267	4.417	36.605
C	33.589	3.946	36.649
C	31.336	3.732	35.816
O	31.877	5.508	37.306
C	33.949	2.797	35.946
H	34.33	4.482	37.243
C	31.717	2.59	35.109
H	30.307	4.098	35.783
C	33.026	2.094	35.164
H	34.98	2.44	36.01
H	30.975	2.05	34.516
C	33.433	0.858	34.396
H	33.95	1.124	33.461
H	32.546	0.262	34.138
H	34.119	0.234	34.986
N	34.006	3.213	44.287
C	33.863	1.879	43.75
C	34.072	3.45	45.615
H	34.057	4	43.629
C	34.989	1.448	42.803
H	32.917	1.78	43.192
H	33.845	1.171	44.585
C	34.24	4.924	45.996

O	33.961	2.589	46.481
N	35.552	2.428	42.062
O	35.319	0.269	42.728
N	34.95	5.73	45.034
H	33.236	5.347	46.145
H	34.77	4.931	46.957
C	36.549	2.131	41.053
H	35.125	3.353	42.052
C	36.301	5.631	44.964
H	34.417	6.185	44.277
H	37.192	3.008	40.907
H	36.091	1.862	40.088
H	37.157	1.284	41.393
C	36.967	6.423	43.859
O	36.952	4.954	45.755
H	37.757	7.048	44.295
H	36.256	7.044	43.299
H	37.443	5.711	43.169
N	19.174	12.288	38.902
C	18.932	13.222	37.981
H	19.919	11.608	38.669
H	18.516	12.114	39.649
N	19.681	13.224	36.875
N	17.98	14.145	38.157
H	20.438	12.528	36.829
H	19.678	14.026	36.257
C	17.11	14.227	39.326
H	17.787	14.761	37.375
C	15.872	13.342	39.224
H	16.823	15.28	39.431
H	17.71	13.983	40.215
H	15.255	13.456	40.125
H	15.269	13.623	38.35
H	16.145	12.281	39.123
C	24.449	3.549	32.083
C	23.78	4.36	30.986
O	25.26	2.657	31.77
O	24.134	3.851	33.287
H	22.689	4.343	31.12
H	24.039	3.977	29.994

H	24.106	5.408	31.07
C	24.068	3.247	37.655
C	23.878	1.817	37.232
N	25.15	3.881	37.256
O	23.21	3.785	38.414
C	23.542	0.993	38.469
H	24.778	1.453	36.719
H	23.048	1.785	36.509
H	25.728	3.447	36.531
H	25.286	4.872	37.507
H	24.314	1.115	39.243
H	22.574	1.302	38.887
H	23.477	-0.074	38.22
N	26.726	0.383	32.726
C	28.166	0.335	32.672
C	26.026	-0.75	32.975
H	26.228	1.257	32.535
C	28.889	0.266	34.021
H	28.53	1.23	32.148
H	28.497	-0.552	32.114
C	24.517	-0.609	32.93
O	26.573	-1.823	33.206
N	28.222	0.774	35.071
O	30.028	-0.196	34.078
H	24.201	0.434	32.798
H	24.098	-1.018	33.858
H	24.137	-1.215	32.096
C	28.782	0.817	36.413
H	27.311	1.205	34.906
C	28.305	2.042	37.182
H	28.505	-0.093	36.973
H	29.878	0.821	36.324
H	28.571	2.947	36.613
H	27.205	2.014	37.248
S	28.994	2.13	38.884
N	21.678	10.434	43.733
C	21.771	10.495	42.392
H	21.501	11.283	44.256
H	21.437	9.563	44.185
N	21.894	11.699	41.804

N	21.731	9.404	41.652
H	22.205	12.478	42.373
H	22.238	11.712	40.84
C	21.534	8.069	42.199
H	21.961	9.481	40.627
C	20.079	7.799	42.579
H	21.862	7.37	41.417
H	22.212	7.916	43.056
H	19.435	7.893	41.694
H	19.714	8.504	43.34
H	19.98	6.781	42.978
N	22.84	4.934	42.893
C	22.998	4.386	41.662
H	22.507	4.325	43.632
H	23.573	5.575	43.184
N	23.908	4.871	40.833
N	22.225	3.361	41.27
H	24.456	5.718	41.016
H	23.825	4.59	39.844
C	20.945	2.97	41.879
H	22.377	3.095	40.294
C	20.85	1.5	42.302
H	20.76	3.629	42.737
H	20.156	3.195	41.145
H	21.6	1.261	43.069
H	19.849	1.309	42.71
H	21.008	0.835	41.442
O	32.225	2.681	39.312
H	32.604	2.532	38.432
H	31.263	2.555	39.151
O	26.147	2.737	34.827
H	26.765	3.497	34.882
H	25.428	3.031	34.218
O	20.796	9.53	35.269
H	21.132	10.099	34.562
H	20.933	10.075	36.08
O	30.057	7.64	37.222
H	29.476	7.554	36.449
H	30.665	6.88	37.146

Large Product Cluster Model (275 atoms, charge -1)

Mg	25.598	10.452	37.004
O	27.694	10.729	37.036
O	25.529	11.908	35.506
H	28.095	10.362	37.847
H	28.116	10.244	36.312
O	25.884	9.145	35.418
O	23.589	10.237	37.079
O	25.82	9.112	38.507
O	25.449	12.079	38.271
H	25.868	12.712	35.924
H	25.864	11.946	34.542
H	26.025	9.514	34.507
H	26.528	8.403	35.486
P	22.541	10.514	38.125
P	25.818	7.626	38.827
P	24.467	12.505	39.317
O	23.059	11.736	39.112
O	21.23	11.031	37.53
O	22.271	9.396	39.13
O	25.11	6.751	37.8
O	25.408	7.32	40.264
O	23.989	14.019	39.098
O	24.838	12.401	40.769
C	23.167	14.284	37.96
H	23.159	15.37	37.814
H	23.574	13.798	37.059
H	22.146	13.92	38.139
C	26.563	11.156	32.368
C	27.02	11.374	30.913
O	26.434	9.983	32.804
O	26.342	12.201	33.053
H	26.295	12.019	30.398
H	27.124	10.421	30.382
H	27.985	11.9	30.922
N	24.708	6.083	35.123
C	23.483	6.602	34.935
H	24.868	5.149	34.751

H	25.171	6.349	36
N	23.136	7.705	35.597
N	22.646	5.996	34.089
H	22.276	8.223	35.406
H	23.848	8.187	36.146
C	21.377	6.576	33.689
H	23.028	5.149	33.629
C	21.526	7.682	32.649
H	20.867	6.966	34.585
H	20.757	5.759	33.299
H	22.201	8.47	33.013
H	20.548	8.135	32.428
H	21.947	7.28	31.716
C	28.262	9.75	40.573
C	28.17	11.213	40.904
O	28.781	9.52	39.368
O	27.903	8.859	41.323
C	28.11	11.462	42.408
H	27.264	11.583	40.387
H	29.025	11.728	40.444
C	28.142	12.936	42.78
H	28.969	10.968	42.886
H	27.21	10.985	42.825
N	26.974	13.638	42.281
H	28.189	13.048	43.875
H	29.056	13.41	42.384
C	26.82	14.96	42.53
H	26.293	13.162	41.685
C	25.602	15.606	41.905
O	27.618	15.592	43.214
H	25.944	16.363	41.185
H	24.957	14.883	41.39
H	25.04	16.129	42.69
N	24.931	9.84	41.737
C	25.128	9.279	42.917
H	24.844	10.854	41.565
H	25.109	9.239	40.922
N	25.033	9.945	44.077
N	25.368	7.938	42.953
C	24.771	11.368	44.277

H	25.222	9.401	44.911
H	25.933	7.619	43.735
H	25.662	7.576	42.028
H	24.058	11.488	45.1
H	24.338	11.795	43.361
H	25.698	11.914	44.505
C	27.703	6.059	35.08
C	27.875	5.988	33.575
O	27.794	7.186	35.658
O	27.505	4.979	35.701
H	27.259	5.183	33.152
H	27.634	6.95	33.106
H	28.93	5.751	33.37
N	22.515	6.66	38.693
C	21.446	6.532	37.893
H	22.69	7.636	38.964
H	23.375	6.21	38.365
N	20.582	7.542	37.83
N	21.246	5.397	37.223
C	19.284	7.519	37.189
H	20.901	8.401	38.29
H	21.844	4.602	37.463
H	20.435	5.277	36.632
C	18.527	8.736	37.679
H	18.755	6.591	37.459
H	19.39	7.564	36.094
H	18.415	8.707	38.773
H	19.075	9.65	37.404
H	17.528	8.781	37.227
N	27.497	7.177	38.618
C	28.013	6.026	39.188
H	28.598	8.581	39.09
H	27.638	7.145	37.597
N	28.221	6.043	40.502
N	28.304	4.997	38.424
C	28.99	5.025	41.213
H	28.078	6.958	40.934
H	28.019	5.03	37.432
H	28.668	4.091	38.8
C	30.471	5.003	40.823

H	28.88	5.255	42.281
H	28.539	4.039	41.029
C	31.113	6.39	40.791
H	30.991	4.351	41.546
H	30.59	4.52	39.841
C	32.641	6.333	40.676
H	30.717	6.954	39.928
H	30.863	6.958	41.701
N	33.04	5.476	39.555
C	33.298	5.914	42.011
H	33.001	7.353	40.473
H	34.061	5.488	39.499
H	32.813	4.496	39.781
H	32.237	5.551	38.158
O	34.019	4.879	42.001
O	33.082	6.646	43.01
C	32.092	4.358	36.615
C	33.425	3.923	36.694
C	31.21	3.661	35.78
O	31.646	5.424	37.323
C	33.843	2.794	35.988
H	34.132	4.467	37.322
C	31.65	2.543	35.069
H	30.172	3.996	35.715
C	32.969	2.077	35.163
H	34.881	2.466	36.085
H	30.943	1.993	34.442
C	33.433	0.858	34.396
H	33.946	1.145	33.465
H	32.572	0.228	34.131
H	34.138	0.261	34.993
N	33.996	3.216	44.282
C	33.863	1.879	43.75
C	34.063	3.458	45.609
H	34.034	4	43.621
C	34.996	1.453	42.81
H	32.919	1.773	43.188
H	33.846	1.173	44.587
C	34.221	4.935	45.984
O	33.962	2.599	46.479

N	35.544	2.43	42.053
O	35.346	0.279	42.752
N	34.936	5.737	45.021
H	33.214	5.355	46.118
H	34.742	4.951	46.949
C	36.549	2.131	41.053
H	35.101	3.349	42.031
C	36.289	5.642	44.964
H	34.397	6.177	44.263
H	37.171	3.02	40.884
H	36.098	1.825	40.095
H	37.177	1.308	41.416
C	36.967	6.423	43.859
O	36.931	4.971	45.766
H	37.79	7.006	44.291
H	36.275	7.083	43.321
H	37.399	5.703	43.149
N	19.195	12.257	38.884
C	18.949	13.199	37.971
H	19.942	11.583	38.642
H	18.521	12.054	39.609
N	19.703	13.219	36.868
N	17.99	14.113	38.151
H	20.464	12.528	36.816
H	19.701	14.03	36.263
C	17.13	14.197	39.328
H	17.798	14.737	37.376
C	15.872	13.342	39.224
H	16.867	15.255	39.451
H	17.732	13.926	40.208
H	15.264	13.46	40.131
H	15.27	13.648	38.358
H	16.12	12.276	39.109
C	24.455	3.536	32.071
C	23.78	4.36	30.986
O	25.29	2.672	31.743
O	24.119	3.799	33.277
H	22.688	4.323	31.112
H	24.053	4.004	29.987
H	24.09	5.411	31.095

C	24.121	3.275	37.741
C	23.993	1.845	37.288
N	25.21	3.947	37.419
O	23.199	3.777	38.444
C	23.542	0.993	38.469
H	24.937	1.497	36.852
H	23.237	1.822	36.486
H	25.844	3.54	36.729
H	25.283	4.951	37.649
H	24.256	1.074	39.303
H	22.553	1.32	38.819
H	23.474	-0.065	38.183
N	26.715	0.407	32.725
C	28.155	0.369	32.674
C	26.027	-0.73	32.987
H	26.21	1.274	32.512
C	28.865	0.273	34.027
H	28.513	1.279	32.173
H	28.496	-0.503	32.1
C	24.517	-0.609	32.93
O	26.587	-1.793	33.236
N	28.224	0.831	35.069
O	29.978	-0.246	34.101
H	24.188	0.427	32.773
H	24.097	-1.001	33.865
H	24.149	-1.24	32.108
C	28.782	0.817	36.413
H	27.321	1.282	34.911
C	28.217	1.938	37.271
H	28.578	-0.152	36.9
H	29.875	0.91	36.335
H	28.406	2.9	36.765
H	27.124	1.818	37.343
S	28.925	1.949	38.966
N	21.645	10.483	43.785
C	21.781	10.51	42.444
H	21.445	11.347	44.274
H	21.341	9.631	44.238
N	21.933	11.697	41.83
N	21.768	9.404	41.729

H	22.191	12.502	42.389
H	22.301	11.693	40.875
C	21.552	8.078	42.29
H	21.976	9.474	40.698
C	20.079	7.799	42.579
H	21.94	7.366	41.546
H	22.175	7.952	43.192
H	19.494	7.869	41.652
H	19.66	8.517	43.3
H	19.96	6.788	42.993
N	23.196	5.054	43.046
C	23.184	4.432	41.843
H	23.057	4.49	43.877
H	23.851	5.825	43.149
N	23.838	4.949	40.819
N	22.459	3.311	41.694
H	24.376	5.828	40.862
H	23.642	4.576	39.873
C	21.728	2.657	42.785
H	22.517	2.861	40.782
C	20.851	1.5	42.302
H	22.447	2.28	43.532
H	21.1	3.417	43.278
H	21.46	0.722	41.821
H	20.34	1.057	43.165
H	20.095	1.854	41.587
O	32.133	2.582	39.339
H	32.497	2.463	38.448
H	31.175	2.412	39.2
O	26.107	2.77	34.902
H	26.732	3.526	34.929
H	25.4	3.032	34.266
O	20.822	9.494	35.268
H	21.152	10.055	34.551
H	20.969	10.047	36.071
O	30.05	7.804	37.274
H	29.383	7.663	36.579
H	30.584	6.988	37.228
